# Graphitic carbon nitride-based photocatalysts for the degradation of contaminants of emerging concern: recent advances and future perspectives

**DOI:** 10.1039/d6ra06963k

**Published:** 2026-10-14

**Authors:** Maryam Ghaffar, Muhammad Saifullah, Mohsin Ali Raza Anjum

**Affiliations:** a Department of Chemistry, Pakistan Institute of Engineering and Applied Sciences (PIEAS) P.O. Nilore Islamabad 45650 Pakistan; b Chemistry Division, Pakistan Institute of Nuclear Science and Technology (PINSTECH) Nilore Islamabad 45650 Pakistan mohsinpieas@hotmail.com +92-51-9248808; c Ulsan National Institute of Science and Technology (UNIST) 50 UNIST-gil Ulsan 44919 South Korea

## Abstract

Contaminants of emerging concern (CECs), such as pharmaceuticals, endocrine disrupting compounds, and personal care products are frequently reported in aquatic environments, which pose serious risks to ecosystems and human health for their potential persistence and resistance to traditional wastewater treatment. Photocatalysis has emerged as one of the most promising sustainable and efficient strategies for degradation of these contaminants. The graphitic carbon nitride (g-C_3_N_4_), as a photocatalyst has gained wide interest owing to its metal-free nature, appropriate bandgap (∼2.7 eV) and excellent chemical stability. However, its practical utilization is restricted by low quantum efficiency and fast charge recombination. This review comprehensively discusses the occurrence and environmental impact of CECs, fundamentals of g-C_3_N_4_ photocatalysis, synthesis approaches, characterization techniques, photocatalytic mechanisms, and recent modification strategies developed to overcome the intrinsic limitations of pristine g-C_3_N_4_. Particular emphasis is placed on the relationship between structural engineering and photocatalytic performance in the degradation of various CECs. The review also discusses the main challenges such as catalyst deactivation, transformation product toxicity, and mass production restrictions. Finally, some emerging directions like AI-guided materials design, green synthesis pathways and pilot-scale practices are discussed that could be explored to close the gap between lab research and real-world applications.

## Abstract 2

This review covers the environmental impact of CECs, g-C_3_N_4_ photocatalysis fundamentals and recent modification strategies to overcome the intrinsic limitations of pristine g-C_3_N_4_.

## Graphical abstract

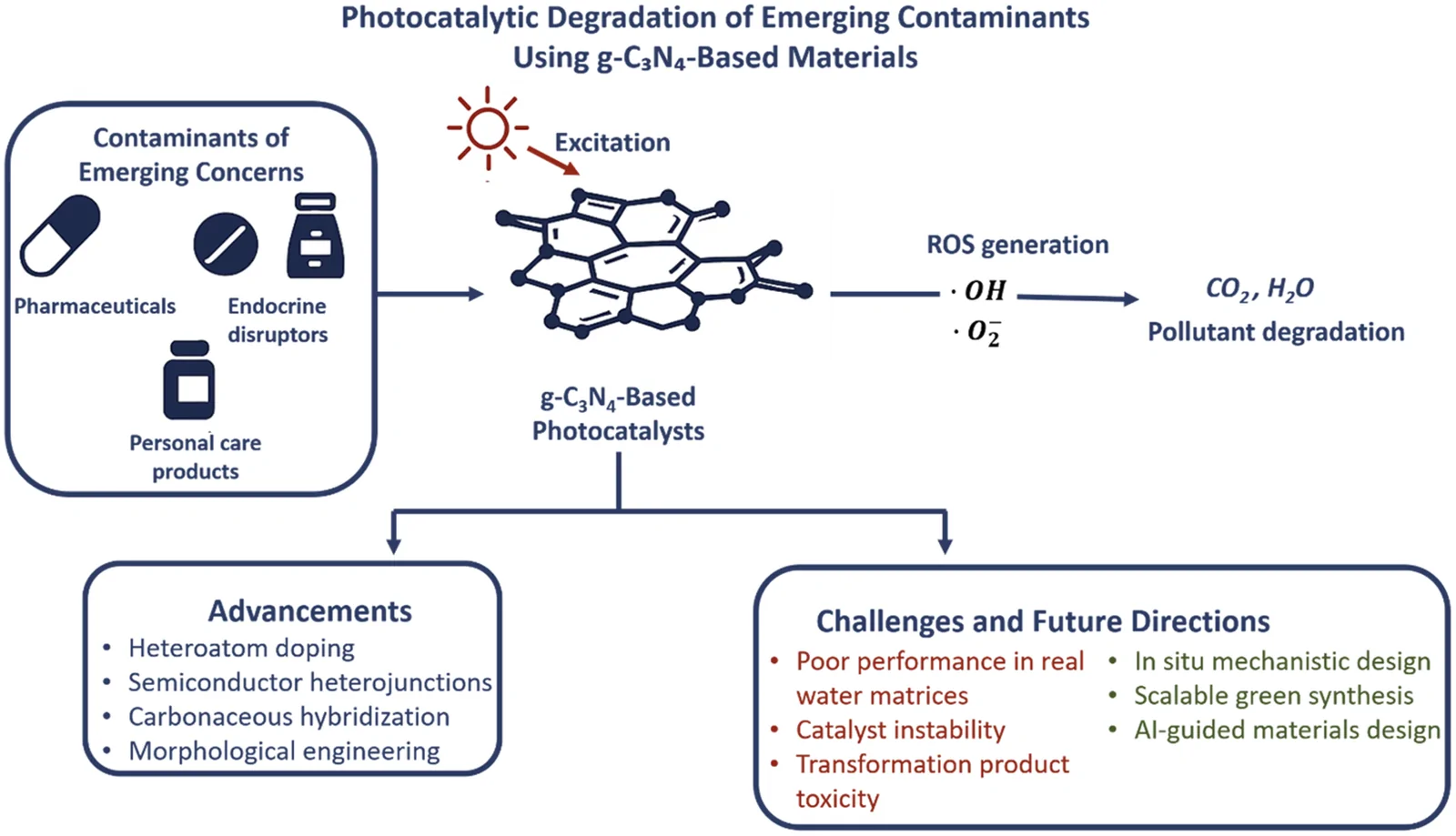

## 1 Introduction

Contaminants of emerging concern (CECs) consist of a wide range of anthropogenic and naturally occurring compounds that are commonly found in water bodies and wastewater effluents. These compounds include pharmaceuticals, personal care products, endocrine-disrupting chemicals, and industrial additives, as well as the persistent transformation by-products which are resistant to conventional wastewater treatments and present bio-accumulative and ecotoxicological responses even at trace levels.^1–4^ From the last decade, the presence of many CECs, such as carbamazepine (CBZ), sulfamethoxazole (SMX), bisphenol A (BPA), and triclosan (TC) have been continuously reported in groundwater, surface water, and even in drinking water reservoirs, which bring serious threats to aquatic ecosystems as well as human health.^5,6^

Conventional wastewater treatment plants (WWTPs) are usually designed for the removal of bulk organic matter, yet they are ineffective in degrading structurally diverse and chemically stable CECs. Approaches like activated sludge and filtration have been proven to be inefficient when it comes to recalcitrant micropollutants, with removal efficiencies often <20% for pharmaceuticals and endocrine disruptors.^7–9^ This operational inefficiency has led to growing attention on advanced oxidation processes (AOPs), and in particular heterogeneous photocatalysis, for solar-driven, low-cost, and eco-friendly degradation pathways by the generation of powerful oxidizing agents such as hydroxyl radicals (˙OH) and superoxide anion (O_2_˙^−^).^10,11^

In the last two decades, among several semiconductor photocatalysts investigated, TiO_2_ has received huge attention compared to other photo-active materials on account of high oxidizing ability and inexpensive nature. Nonetheless, its large band gap (∼3.2 eV) limits the sunlight absorption to ultra-violet (UV) region (<5% of the solar spectrum), and in addition, its photocatalytic performance is greatly suppressed due to rapid recombination rate between photo-generated electrons and holes.^12–14^ Similarly, metal oxides-based materials, such as ZnO and SnO_2_ possess strong stability, but can only be activated under UV light.^15,16^ Bismuth-based materials (*e.g.*, BiVO_4_, Bi_2_WO_6_) and silver halides often suffer from photo-corrosion and metal-leaching issues.^17–19^ Perovskites and MOFs exhibit modifiable structures and large surface areas, but their hydrothermal instability and high cost prevent successful scaling up.^20–22^ Among them, g-C_3_N_4_ has been widely researched because of its natural abundance, metal-free character, moderate band gap (∼2.7 eV), adjustable structure and chemical stability under irradiation and various pH conditions.^23–25^

The g-C_3_N_4_ photocatalyst was first prepared by thermal polycondensation of nitrogen-rich precursors, melamine and urea, which formed a two-dimensional (2-D) network, where conjugated heptazine or triazine units linked to each other *via* covalent bonds.^26,27^ Wang *et al.* were the first to introduce a metal-free, visible-light photocatalyst for hydrogen evolution in the form of polymeric g-C_3_N_4_, and shortly afterwards Yan *et al.* (2009) proved its environmental importance with the photodegradation of methyl orange, organic azoic model dye pollutant.^28,29^ Nonetheless, pristine g-C_3_N_4_ has various drawbacks, such as fast recombination of the electron–hole pair, small specific surface area, narrow redox potential range, and weak interactions with pollutant molecules which limits its practical applications.^30–33^

To solve these bottlenecks, many strategies that involve modification have been explored, such as elemental doping, construction of heterojunctions, nano-structuring, interfacial engineering, and hybridization with the carbonaceous materials.^34–39^ The purpose of these modifications is to broaden the light absorption in the visible region, improve charge separation, expose more surface-active sites, and enhance adsorption capacity and selectivity toward target pollutants. The obtained g-C_3_N_4_-based composites exhibited improved photocatalytic performance under visible light for degradation of various CECs, including fluoroquinolone antibiotics (*e.g.*, mpg-C_3_N_4_/CQDs) and phenolic derivatives (*e.g.*, CDs/g-C_3_N_4_ for BPA).^4,36,40^

Despite extensive studies, only few systematic reviews focused on g-C_3_N_4_-based photocatalytic removal of CECs compared to exhaustively covered TiO_2_.^41^ The majority of the already available reviews are either too wide in scope (*e.g.*, like general photocatalytic applications), or are specifically around material design without representing CEC-oriented performance or mechanistic gaps.^36,42^

The aim of the present review is to fill this important gap in knowledge, through a detailed and mechanistic-oriented review of g-C_3_N_4_ as a photocatalyst for CECs degradation. This review focuses specifically on the degradation of CECs. It connects the properties of CECs with suitable g-C_3_N_4_ modification strategies and discusses the associated mechanistic trade-offs. Particular attention is given to transformation pathways, intermediate toxicity, and mineralization, which are often less discussed than parent-pollutant removal. The review also considers practical issues such as real-water performance, catalyst stability, continuous-flow operation, and standardized experimental reporting. Recent developments in AI-assisted catalyst design and single-atom catalysts are also discussed to provide an updated perspective on future g-C_3_N_4_-based treatment systems.

## 2 Contaminants of emerging concern (CECs)

Over the past few decades, the term “contaminants of emerging concern” (CECs) has gained significant importance in environmental science to describe a broad and dynamic class of synthetic and naturally occurring chemicals, that are increasingly being found in aquatic ecosystems, but remain largely unregulated.^43^ These compounds are often present in trace amounts (ng L^−1^–µg L^−1^), but their high persistence and bio-accumulative nature make them difficult to remove by conventional water and wastewater treatments.^44–46^ Their long-term exposure, even at low concentrations, have been linked to oxidative stress, DNA damage, and disturbance of microbial communities in aquatic ecosystems.^47–49^

Additionally, transformation products formed during photolysis, chlorination or biodegradation might be more toxicologically active and persistent than their parent compounds, yet they are rarely monitored or regulated.^50,51^ For example, the chlorination of triclosan led to the formation of dioxins under UV exposure, raising additional concerns in treated water supplies.^52^ Similarly, the photodegradation of sulfamethoxazole (SMX) produced sulfanilic acid and other aromatic amines with known cytotoxicity.^53^

As summarized in Table 1, a wide range of compounds are classified as contaminants of emerging concern (CECs). These include, flame retardants, surfactants, complexing agents, organic solvents such as methyl tertiary-butyl ether and ethyl tertiary-butyl ether, per-fluorinated compounds, engineered nanoparticles, artificial sweeteners, drugs, a variety of pharmaceuticals and personal care products, gasoline derivatives and their transformation products, biocides, polar pesticides and their by-products, as well as various endocrine-disrupting chemicals.

**Table 1:** Representative classes and subclasses of CECs along with examples and their applications

Classes/sub classes	Examples	Uses
**Artificial sweeteners**		
Artificial sweeteners	Alitame, dulcin, stevioside, neotame, rebaudioside A, aspartame, saccharin	Sugar substitute

**Endocrine disrupting substances**		
Steroids	Estradiol, testosterone	Hormone therapy, hormone replacement
Alkyl phenols	Nonylphenol ethoxylate, 4-nonylphenol	Textile processing, emulsifying agent
Polyaromatic compounds	Perylene, fluorene, anthracene	Organic semiconductor, optical brightener, dye production
Organic oxygen compounds	Resorcinol, BPA	Hair dye, epoxy resins

**Flame retardants**		
Halogen-containing flame retardants	Polybrominated diphenyl ethers, chlorinated paraffins	Furniture foam, PVC materials
Borate flame retardants	Borax, calcium borate, barium borate	Wood treatment, optical materials
Silicon flame retardants	Polydimethylsiloxane, silicone resins, siloxane oligomers	Cable insulation, coating protection, fire-resistant foams
Inorganic hydroxide flame retardants	Mixed metal hydroxide, calcium hydroxide, hydrotalcite	Flame-retardant fillers, polymer stabilizer, PVC stabilizer
Phosphorus-based flame retardants	Triphenyl phosphate, tris(2-chloroethyl) phosphate	Plasticizer, PVC, flexible foams

**Pesticides**		
Triazines	Atrazine (ATN), simazine, propazine, cyanazine, terbutryn, triasulfuron	Herbicide, algae control, insecticide
Carbamates	Carbaryl, carbofuran, methomyl, aldicarb	Insecticide, nematicide
Organochlorines	Dieldrin, endrin, heptachlor	Termite control, insecticide, seed treatment, mosquito control
Chloroacetanilides	Alachlor, propachlor, pretilachlor	Herbicide, weed control
Pyrethroids	Permethrin, cypermethrin, fluvalinate	Mosquito control, crop insecticide, fruit pest control

**Pharmaceuticals and personal care products**		
Antimicrobial	TC, triclocarbon, benzalkonium chloride	Antibacterial soap, body wash, hand sanitizer
Analgesic non-steroidal anti-inflammatory drugs	Ibuprofen, naproxen, diclofenac (DCF), ketoprofen	Pain relief, arthritis treatment
Synthetic hormones	Estradiol valerate, prednisone, hydrocortisone	Hormone therapy, allergy treatment, skin inflammation
Polycyclic musks	Galaxolide, tonalide, traseolide	Perfume ingredient, scent fixative, body spray

Pharmaceuticals such as DCF, CBZ, ciprofloxacin (CIP), and SMX are broadly used in both human and veterinary medicines. These compounds, along with their transformation products, are frequently detected in WWTP effluents at concentrations ranging from tens to hundreds of ng L^−1^.^54,55^ Similarly, endocrine disruptors like BPA, 17β-estradiol, and nonylphenol mimic hormonal activity even at low concentrations and are associated with reproductive and developmental toxicity in aquatic organisms.^56,57^ Flame retardants (*e.g.*, polybrominated diphenyl ethers), UV filters (*e.g.*, benzophenone derivatives), and synthetic musk in personal care products have also been shown to exert chronic toxicity, endocrine disruption, and genotoxic effects.^58,59^

Numerous national and international monitoring efforts have confirmed the widespread occurrence CECs in estuarine systems, surface water, ground water, and even in drinking water sources. For instance, a global meta-analysis by Wilkinson *et al.* (2022) covering 105 countries found at least one CEC in over 92% of the sampled surface waters, with CBZ, caffeine, and metformin being the most frequently detected.^4^ The European Union (EU) maintains a watch list of emerging contaminants under the Water Framework Directive, and recently updated it to include pharmaceuticals and UV filters, while the United States Environmental Protection Agency (USEPA), periodically updates its Contaminant Candidate List (CCL) with the latest edition (2022) listing 66 chemicals and 12 microbes.^60,61^Table 2 summarizes Environmental Quality Standards (EQS) for different CECs along with respective regulatory authority.

**Table 2:** Representative regulatory limits and EQS for selected CECs in water

Contaminants	Regulatory source	EQS/limits (µg L^−1^)	Ref.
DCF	EU (2013)	0.1 (surface water), 0.01 (coastal surface water)	62
Carbamazepine	EU draft (proposed, 2022)	0.54 (drinking water)	63
Total per- and polyfluoroal alkyl substances (PFAS)	EU (2020)	0.5 (drinking water)	64
ATN	WHO (2022)	100 (drinking water)	65
17α-Ethinylestradiol (EE2)	EU/JRC draft (proposed, 2011; reiterated 2018)	0.000035 (surface water)	66
17β-Estradiol (E2)	JRC draft (proposed, 2018)	0.0004 (surface water)	67
BPA	EU (2020)	2.5 (drinking water)	68
Triclosan	Federal Water Quality Guideline (FWQG, 2017)-Canada	0.47 (fresh water aquatic life)	69

Conventional WWTPs such as coagulation, membrane filtration, and activated sludge are often ineffective in eliminating trace-level CECs. As a result, research attention has now shifted toward AOPs, which can mineralize persistent pollutants through highly reactive oxygen species (ROS). The following section elaborates on the working principles, limitations, and photocatalytic advances within AOPs, with particular focus on g-C_3_N_4_ systems.

## 3 Advanced oxidation processes (AOPs) for CECs removal

AOPs have been recognized to be the one of the most efficient methods for CECs removal, as they produce highly ROS, which are capable of non-selectively oxidizing a huge number of organic contaminants.^70,71^ AOPs are a range of processes, which include ozonation, Fenton and photo-Fenton systems, UV/H_2_O_2_ processes, sulfate radical (SO_4_˙^−^) based processes and semiconductor photocatalysis.^72^ These systems rely on the *in situ* generation of radicals such as ˙OH, O_2_˙^−^, and SO_4_˙^−^ radicals, which initiate oxidation reactions with persistent and non-biodegradable pollutants.^73–75^ Due to their unmatched oxidative potential (˙OH: 2.8 V, SO_4_˙^−^: 2.6 V *vs.* NHE), these species are capable of transforming CECs into smaller, less toxic molecules or achieving full mineralization into CO_2_ and H_2_O.^76,77^

However, many conventional AOPs suffer from critical drawbacks that limit their large-scale application. Fenton and photo-Fenton reactions lead to the production of iron rich sludge, require acidic pH (∼3), and are susceptible to inorganic ions that can serve as radical scavengers.^78^ Similarly, ozonation, though effective for aromatic and electron-rich compounds, is expensive and can produce ozonation by-products such as aldehydes, and carboxylic acids, which may be more toxic than their parent compounds.^79^ UV-based AOPs like UV/H_2_O_2_ depend heavily on artificial light sources, increasing energy consumption, and are less efficient under real sunlight because of the inherent narrow quantum yield and recombination of photo-induced carriers.^80^

In this context, photocatalysis driven by solar energy represents a sustainable and attractive AOP pathway, especially when utilizing visible-light-responsive photocatalysts. Semiconductor photocatalysis involves light absorption to excite electrons from the valence band (VB) to the conduction band (CB), generating photogenerated electron–hole pairs that take part in redox reactions at the catalyst surface.^14,81^ The efficiency of such systems depends on many key factors, including type of photocatalyst (surface area, surface charge, concentration, composition, defects, light absorption range, charge separation efficiency), light source, (intensity, wavelength, distance), reaction medium (pH related to target contaminant, solvent), recombination of photoinduced electron–hole pairs, and redox potential alignment.

Among these, graphitic g-C_3_N_4_ has emerged as promising photocatalysts for CECs degradation. The g-C_3_N_4_ exhibits a moderate bandgap, allowing it to absorb up to 460 nm of solar light, with a VB potential of ∼1.4 V (*vs.* NHE) and a CB of ∼−1.3 V (*vs.* NHE).^82^ Moreover, its metal-free composition, thermal stability, and tunable electronic structure make it highly suitable for environmental applications. The g-C_3_N_4_ can activate molecular oxygen and water molecules under visible light to generate ROS without requiring additional reagents or harsh conditions, distinguishing it from traditional AOPs.^83–85^

Recent research has confirmed the effectiveness of g-C_3_N_4_-based photocatalysts in degrading a wide array of CECs, including antibiotics (*e.g.*, SMX, ciprofloxacin), personal care products (*e.g.*, triclosan, benzophenone-3), and pharmaceuticals (*e.g.*, carbamazepine, naproxen) under visible or simulated solar irradiation.^86,87^ For example, studies have reported that appropriately modified g-C_3_N_4_ can achieve high degradation efficiency of antibiotics, for instance, over 90% removal of SMX within one hour of irradiation.^88^ Visible light driven g-C_3_N_4_ based heterojunctions photocatalysts have achieved efficient degradation of diverse pharmaceuticals while simultaneously yielding transformation products of lower acute and ecological toxicity, in contrast to UV/H_2_O_2_ systems, which are frequently associated with the formation of toxic by-products.^89,90^

Although AOPs can produce various reactive species, such as solvated electrons and hydrogen atoms, ˙OH remains the most potent and non-selective oxidants.^91,92^ With a standard redox potential ranging from 1.9 to 2.7 V *vs.* NHE depending on pH, ˙OH radicals are highly effective in degrading organic contaminants.^93,94^ Their efficient generation, particularly through g-C_3_N_4_-based heterogeneous photocatalysis, has resulted in extensive research into their use for removing CECs from water.^95^

Another advantage is the potential of g-C_3_N_4_ to function synergistically with other treatment methods. Coupling photocatalysis with biological processes, membrane filtration, or electrochemical oxidation has shown promising results in enhancing overall CEC removal and reducing treatment time. For instance, photocatalytic pre-treatment has enabled comprehensive removal in intimately coupled photocatalysis-biodegradation systems, while g-C_3_N_4_-based visible-light responsive membranes deliver simultaneous filtration, degradation and antifouling.^96,97^Fig. 1 and eqn (1)–(8) illustrate the important reactions involved in the g-C_3_N_4_-driven photocatalytic degradation of organic CECs. The degradation of the organic CEC compounds results in the formation of carbon dioxide, water, and other organic byproducts, including nitrogenous compounds, as final products of mineralization.

1 *E*_photon_ = *hν*

2 g-C_3_N_4_ + *E*_photon_ → h_VB_^+^ + e_CB_^−^

3 h_VB_^+^ + CEC → Oxidized intermediates

4 e_CB_^−^ + O_2_ → O_2_^˙−^

5 O_2_^˙−^ + H^+^ → HOO^˙^

6 2HOO^˙^ → H_2_O_2_ + O_2_

7 H_2_O_2_ + e_CB_^−^ → HO^˙^ + OH^−^

8 HO^˙^, O_2_^˙−^, and other ROS + CECs → Degraded products + CO_2_ + H_2_O

In eqn (1), *h* represents Planck's constant, and *ν* denotes the frequency of the incident photons. When the photons of energy equal to or higher than the band gap of g-C_3_N_4_ irradiate the material (eqn (1)), electrons are excited from VB to CB, leaving behind electron deficient holes (h^+^) in the VB (eqn (2)). These photogenerated h^+^ can directly oxidize CEC molecules into oxidized intermediates (eqn (3)), while the excited electrons reduce surface-adsorbed oxygen to form O_2_˙^−^ (eqn (4)). The O_2_˙^−^ can react with H^+^ to form hydroperoxyl radicals (˙OOH), which further produce hydrogen peroxide (H_2_O_2_) (eqn (5) and (6)).^98–100^ H_2_O_2_ can then react with photogenerated electrons to generate ˙OH radicals (eqn (7)). These ROS (˙OH and O_2_˙^−^) initiate the degradation of organic pollutants into smaller organic molecules, water and CO_2_ (eqn (8)).^101,102^

**Fig. 1:**
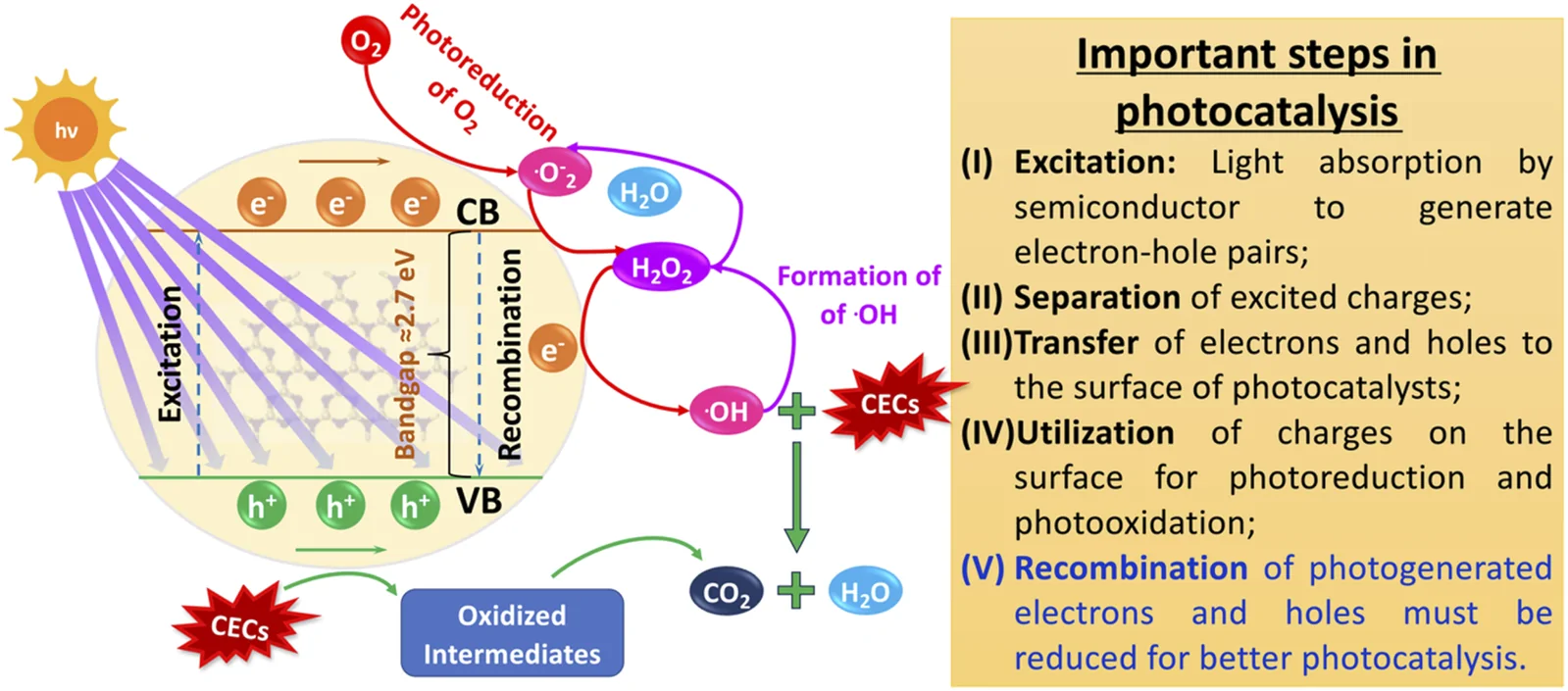
Schematic illustration of the important steps involved in the photooxidation of CECs and photoreduction of O_2_ over a semiconductor g-C_3_N_4_ photocatalyst.

## 4 Fundamentals of g-C_3_N_4_ photocatalysis

The history of carbon nitride dates back to 1834, when Berzelius first synthesized it in a laboratory by pyrolysis of mercuric thiocyanate.^103^ Subsequently, Liebig synthesized melem, melam, and melamine (Fig. 2a–c), *via* thermal pyrolysis of potassium thiocyanate and ammonium chloride and reported that these compounds could further condensed into triazine- and heptazine-based molecular compounds. The resulting yellow polymeric material was named “melon” (Fig. 2d).^104^

**Fig. 2:**
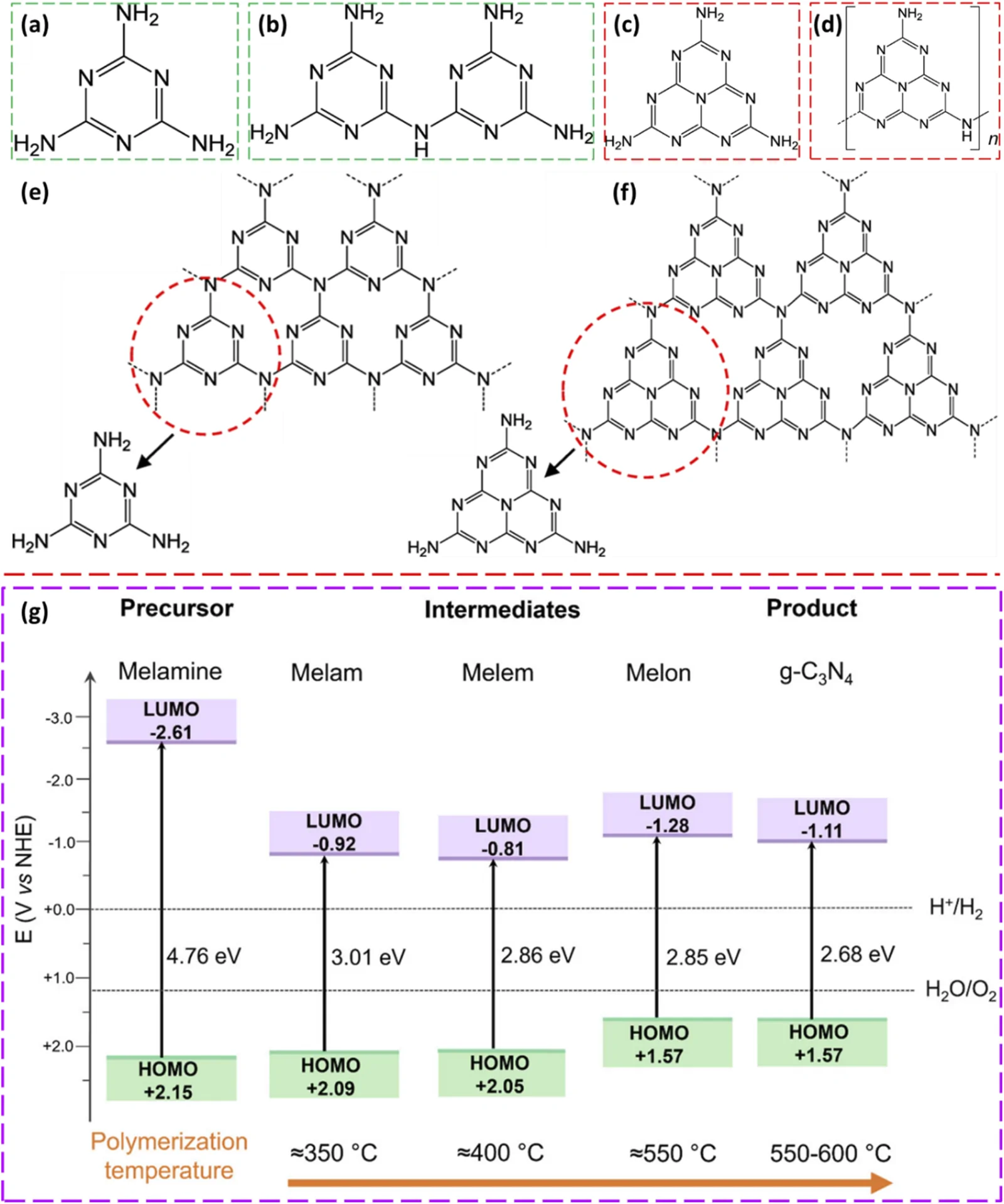
Chemical structure of: (a) melamine, (b) melam, (c) melem, and (d) melon as documented by Liebig; schematic structural models of g-C_3_N_4_: (e) triazine-based network; (f) tri-*s*-triazine (heptazine) network, reproduced with permission from ref. 109 and 110 Copyright 2008 Royal Society of Chemistry and Copyright 2016 American Chemical Society; (g) band gap, HOMO–LUMO positions of precursor (melamine), intermediates (melam, melem, melon), and the product (g-C_3_N_4_) formed during the polymerization reaction, reproduced with permission from ref. 112. Copyright 2023 American Chemical Society.

The g-C_3_N_4_ polymeric semiconductor, primarily composed of carbon (C) and nitrogen (N) atoms, has emerged as one of the most extensively investigated visible-light active photocatalyst, including hydrogen evolution, CO_2_ reduction, and more recently, degradation of CECs.^105–107^ This section will provide an in-depth discussion of structural and electronic properties, synthesis strategies, photocatalytic mechanisms, surface characteristics, and charge-carrier dynamics of g-C_3_N_4_-based photocatalysts relevant to environmental remediation applications.

### 4.1 Structure and phases of g-C_3_N_4_

g-C_3_N_4_ is generally described as a layered polymeric semiconductor, constructed from triazine or heptazine (tri-*s*-triazine) repeating units connected through planar C–N linkages.^108^ These extended π-conjugated networks form graphitic sheets stacked together by van der Waals forces, as illustrated in Fig. 2e and f.^109,110^ The delocalized π-electron system across the heptazine rings enables efficient charge migration, while interlayer stacking facilitates exciton transport between adjacent planes.^111^ Structural variations between the triazine- and heptazine-based motifs (Fig. 2e and f), influence crystallinity, electronic band dispersion, and overall photocatalytic performance. During the polymerization of melamine, the intermediates melam, melem, and the product melon are formed at 350, 400 and 500 °C, accompanied with the band gap decreases from 4.76 eV to 2.68 eV. The band energy diagram (Fig. 2g) illustrates the band gap variation with increasing the degree of polymerization of melamine to intermediates and final product g-C_3_N_4_.^112^

The first-principles density functional theory (DFT) calculations, have predicted the five polymorphs of carbon nitride polymer, including alpha (α), beta (β), cubic, pseudocubic, and graphitic phases.^113^ The β-C_3_N_4_ phase has identical structure to phenacite (Be_2_SiO_4_), belongs to trigonal *P*3 space group and contains 14 atoms per unit cell (Fig. 3a). The α-C_3_N_4_ phase adopts *P*3_1_*c* symmetry with 28 atoms per unit cell. Its structure can be described as an ABAB… stacking sequence of β-C_3_N_4_-type layers and its mirror counterpart (Fig. 3b). The g-C_3_N_4_ phase crystallizes in the hexagonal *P*6̄*m*2 space group with 14 atoms per unit cell. It consists of planar layers arranged in an ABAB… stacking sequence of graphitic system (Fig. 3c). The proposed configuration for pseudocubic-C_3_N_4_ (pc-C_3_N_4_) is suggested similar to α-CdIn_2_Se_4_ defect-zinc blende type structure. This belongs to the *P*4̄2*m* space group and contains seven atoms per unit cell (Fig. 3d). As shown in Fig. 3e, the cubic-C_3_N_4_ (c-C_3_N_4_) exhibits the willemite-II structure type (Zn_2_SiO_4_), it adopts cubic *I*4̄3*m* symmetry upon structural relaxation.^113^ These DFT calculations also predict that α-C_3_N_4_ and g-C_3_N_4_ are energetically favoured relative to β-C_3_N_4_. However, the tri-*s*-triazine-based g-C_3_N_4_ is the most stable one carbon nitride.

**Fig. 3:**
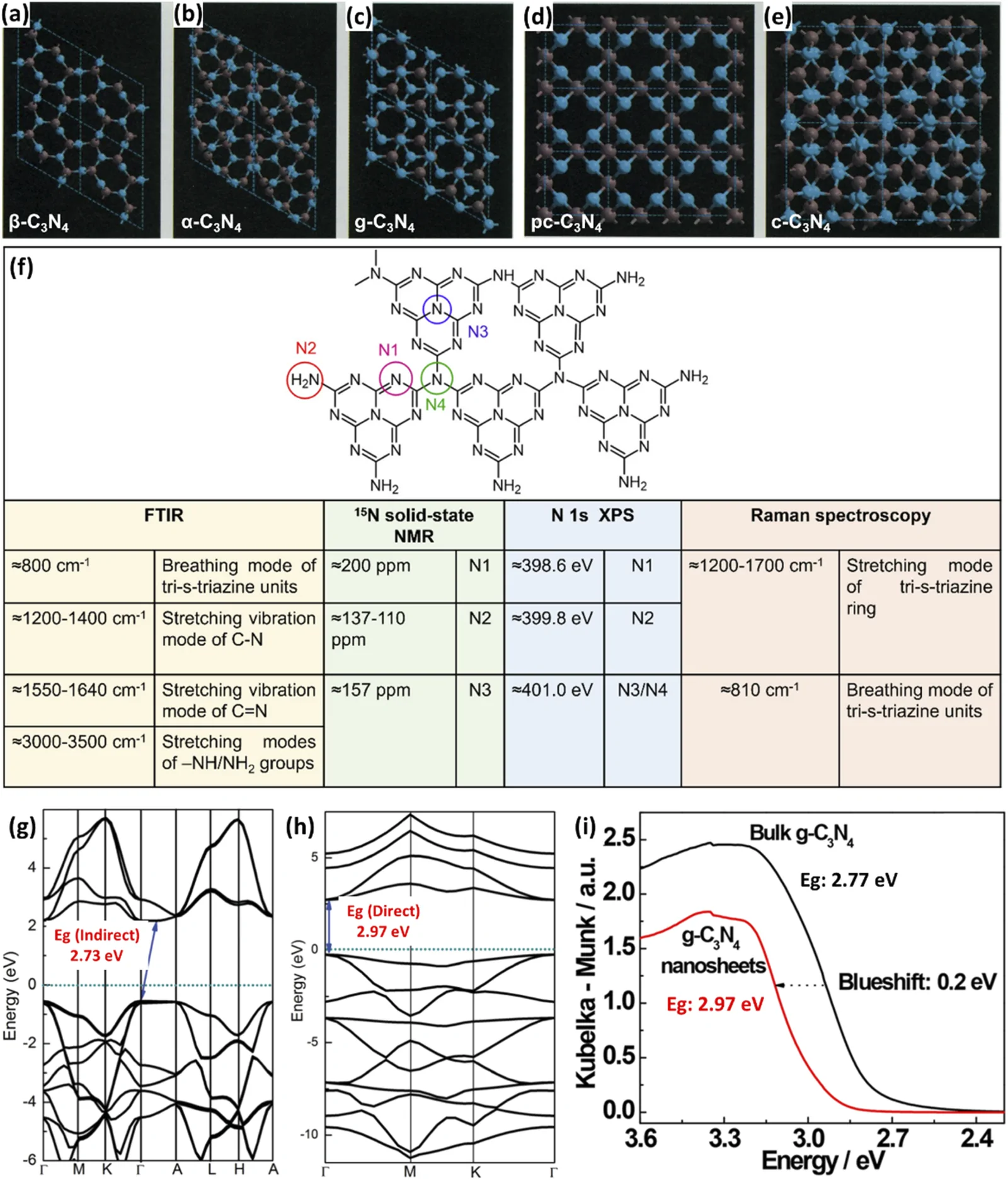
Theoretically predicted structure of (a) β-C_3_N_4_, (b) α-C_3_N_4_, (c) g-C_3_N_4_, (d) pc-C_3_N_4_, and (e) c-C_3_N_4_, C and N atoms are shown as grey and blue spheres, respectively, reproduced with permission from ref. 113 Copyright 1996 American Association for the Advancement of Science; chemical structure of (f) tri-*s*-triazine-based g-C_3_N_4_ showing the N1 (pink), N2 (red), N3 (purple), and N4 (green) atoms involved in bonding along with summary of experimental evidences derived from FTIR, ^15^N solid-state NMR, XPS, and Raman spectroscopy, reproduced with permission from ref. 112; calculated band structures/gaps of: (g) bulk (indirect *E*_g_) and (h) monolayer g-C_3_N_4_ (direct *E*_g_) using HSE06 hybrid functional g-C_3_N_4_, reproduced with permission from ref. 115 Copyright 2016 Royal Society of Chemistry; (i) the experimentally calculated direct and indirect band gap of bulk g-C_3_N_4_ and g-C_3_N_4_ nanosheets using UV-visible DRS absorption spectra, reproduced with permission from ref. 117 Copyright 2014 Royal Society of Chemistry.

Fig. 3f shows two sets of nonequivalent N atoms in the tri-*s*-triazine unit, the N1 (pink) is coordinated with two atoms, while N2 (red), N3 (purple) and N4 (green) are coordinated three atoms. The chemical and electronic properties of tri-*s*-triazine-based g-C_3_N_4_ are also investigated by Fourier transform infrared (FTIR), different stretching modes of functional groups, such as –C–N, –C=N and –NH/–NH_2_ are observed in the ranges of ∼1200–1400, ∼1550–1640 and ∼3000–3500 cm^−1^ respectively. Raman spectroscopy shows the breathing mode at ∼800 and stretching mode in the range of ∼1200–1700 cm^−1^, the ^15^N solid state-nuclear magnetic resonance (NMR) confirms the N1 (∼200 ppm), N2 (∼137–110 ppm) and N3 (∼157 ppm) and X-ray photoelectron spectroscopy (XPS) determines the binding energies for N1, N2, N3 and N4, as summarized in Fig. 3f.^112^

### 4.2 Electronic characteristics of g-C_3_N_4_

The sp^2^ hybridization of C and N atoms in the conjugated g-C_3_N_4_ system is mainly responsible for the material's UV-visible light absorption properties. The C and N atoms are connected in the tri-*s*-triazine units through strong sigma bonds between sp^2^–sp^2^ hybrid orbitals, which creates sheet-like graphitic structure, while unhybridized half-filled p_*z*_ orbitals remain perpendicular to the graphitic plane across the whole g-C_3_N_4_ system. These p_*z*_ orbitals overlap with each other to form π (bonding) and π* (antibonding) energy bands. First-principles calculations also show that the HOMO is mainly composed of N 2p and 2s orbitals, whereas the LUMO primarily consists of 2p orbitals of C and N atoms responsible for π* interactions.^114^

The intrinsic electronic structure of g-C_3_N_4_ governs its light absorption, redox capability, and overall photocatalytic activity. Bulk g-C_3_N_4_ is an indirect band gap semiconductor while monolayer g-C_3_N_4_ nanosheet is direct gap semiconductor material. The theoretically calculated band gap of bulk g-C_3_N_4_ (2.73 eV) is much less than monolayer g-C_3_N_4_ (2.97 eV), as shown in Fig. 3g & h.^115^ These calculated band gaps are well matched with the experimentally determined values of ∼2.70 to 2.77 eV for bulk and 2.97 eV for nanosheets as shown in UV-visible DRS absorption spectra (Fig. 3i).^116–118^ The indirect bandgap for bulk g-C_3_N_4_ (∼2.70 eV) corresponds to an absorption edge of ∼460 nm, which enables visible-light activation under natural solar irradiation.^31,119^ The VB primarily comprises of 2p orbitals of N atoms, while the CB arises from 2p orbitals of C atoms, yielding a VB potential of approximately +1.4 V and a CB potential of −1.3 V *vs.* NHE, at pH 7. The negative CB potential of g-C_3_N_4_ well within the window for O_2_˙^−^, whereas the VB of pristine g-C_3_N_4_ is generally insufficient for direct ˙OH from H_2_O/OH^−^. Therefore, ˙OH detected in g-C_3_N_4_ systems may originate through secondary ROS pathways, including H_2_O_2_ formation, or thorough band-edge modification in heterostructure photocatalysts.^31,120^

#### 4.2.1 Different strategies to extend optical absorption of g-C_3_N_4_

Two types of electronic transitions are theoretically possible in g-C_3_N_4_, *i.e.* the more photoactive π → π* and much less photoactive n → π*. When the light interacts with g-C_3_N_4_, the fundamental optical transition (T_1_) involves excitation of electrons from the π (HOMO) to π* (LUMO) orbitals, resulting in an absorption edge at ∼460 nm as shown in Fig. 4a. There are two possible transitions, *i.e.* the lone-pair electrons of edge N atoms (HOMO) to π* orbitals (n → π*; called T_2_) and from π (HOMO) to N vacancies (π → n*; T_3_). The T_2_ transitions are predicted to produce a shoulder at wavelengths ≥490 nm. However, such transitions (T_2_ and T_3_) are rarely observed in planar, symmetric g-C_3_N_4_ because the lone-pair orbitals are orthogonal to the π-conjugated plane and are not efficiently photoexcited. Consequently, π → π* transitions are solely accountable for visible-light absorption, yielding a band gap of approximately 2.75 eV.

**Fig. 4:**
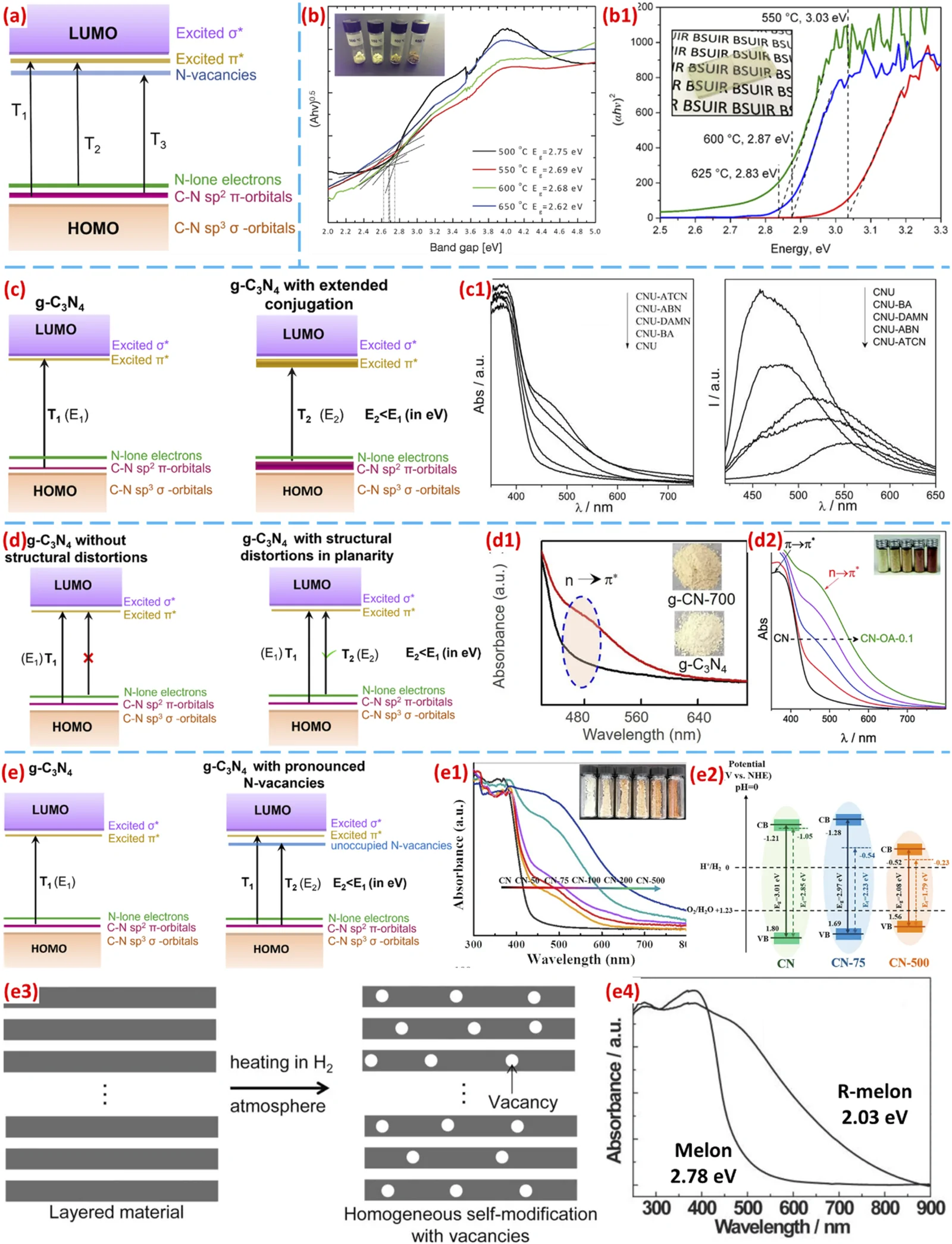
(a) Band gap energy (*E*_g_) diagram of g-C_3_N_4_ showing fundamental electronic transition (T_1_: π → π*) and possible transitions (T_2_: n → π* & T_3_: π → n*), reproduced from ref. 112 with permission from American Chemical Society, Copyright 2023; Tauc plots of g-C_3_N_4_ (b) powder synthesized at 500, 550, 600, and 650 °C, adopted from ref. 121 with permission from Royal Society of Chemistry, Copyright 2025; (b1) thin films prepared on glass substrate at 550, 600, and 625 °C, reproduced from ref. 122 with permission from American Chemical Society, Copyright 2022; (c) suggested changes in energy band due to degree of conjugation by copolymerization, reproduced from ref. 112 with permission from American Chemical Society, Copyright 2023; (c1) UV-visible DRS spectra and PL spectra of copolymerized g-C_3_N_4_ (CNU) samples (here, BA: barbituric acid, DAMN: diaminomaleonitrile, ABN: aminobenzonitrile, ATCN: 2-aminothiophene-3-carbonitrile), adopted from ref. 123 with permission from Royal Society of Chemistry, Copyright 2014; (d) energy band diagram of n → π* enabled transitions due to structural distortions in planarity, reproduced from ref. 112 with permission from American Chemical Society, Copyright 2023; (d1) UV/vis DRS spectra for pristine and annealed g-C_3_N_4_ at 700 °C/20 min (g-CN-700), adopted from ref. 124 with permission from Elsevier B.V., Copyright 2020; (d2) pristine (CN) and oxamide-modified g-C_3_N_4_ (CN-OA), adopted from ref. 125 with permission from Royal Society of Chemistry, Copyright 2017; (e) comparison of energy band diagram of g-C_3_N_4_ without and with N-vacancies, reproduced from ref. 112 with permission from American Chemical Society, Copyright 2023; (e1) UV-vis spectra of CN (g-C_3_N_4_ prepared from urea) and CN-*x* synthesized from mixture of urea and N_2_H_4_·H_2_O (*x* = 50 µg, 75 µg, 100 µg, 200 µg and 500 µg) and (e2) band structure description of CN, CN-75 and CN-500, reproduced from ref. 126 with permission from Elsevier B.V., Copyright 2020; (e3) homogeneous self-modification strategy to produce vacancies in a layered g-C_3_N_4_ by reducing in a H_2_ atmosphere, (e4) comparison of UV-visible absorption spectra of melon and R-melon, reproduced from ref. 127 with permission from John Wiley & Sons, Inc., Copyright 2014.

Despite its promising potential for photocatalytic applications, further enhancement of its light-harvesting ability beyond ∼460 nm always remains challenge, as pristine g-C_3_N_4_ perform well in visible light (≤460 nm) due to T_1_ transitions only, corresponding to ∼10% of the solar spectrum. Therefore, narrowing its band gap to capture a broader portion of sunlight is essential for improving its solar energy conversion efficiency. To achieve this, various modification strategies have been systematically investigated, including (i) increasing the degree of polymerization at high temperature, (ii) degree of conjugation by copolymerization of precursors (melamine, urea dicyandiamide) with other organic comonomer molecules (*e.g.* 2-aminobenzimidazole, 2-aminoterephthalic acid, *etc.*) (iii) structural distortion of the planar framework (*e.g.* porous ultrathin g-C_3_N_4_ nanosheets), (iv) introduction of nitrogen vacancies, and (v) tuning the C/N ratio.^112^

As discussed earlier in Section 4.1 (Fig. 2g), the degree of polymerization significantly reduces the band gap. The experimental and theoretical investigation has shown the direct relationship between band gap and structural aspects. As shown in Fig. 4b, the band gap reduces from 2.75 eV to 2.62 eV with increasing the degree of polymerization by elevating the temperature from 500, to 650 °C, under Ar gas atmosphere.^121^ In another study, the transparent film of g-C_3_N_4_ was grown on glass substrates at different temperatures. The optical band gap of the g-C_3_N_4_ films linearly decreases with the rise in synthesis temperature from 550 °C (3.03 eV) to 625 °C (2.83 eV), as extracted from Tauc plot (Fig. 4b(b1)).^122^

Copolymerization of starting precursors for g-C_3_N_4_ with another comonomers such as, barbituric acid (BA), diaminomaleonitrile (DAMN), aminobenzonitrile (ABN), 2-aminothiophene-3-carbonitrile (ATCN), is another method to improve the optical absorption. As discussed in literature, the copolymerization is an optimistic strategy to extend the light absorption (or reduced band gap) by enhancing the degree of conjugation without compromising the structural integrity of g-C_3_N_4_. This approach also assists to increase the density of state (DOS) at HOMO and LUMO bands, resulting in a significant π → π* electronic transition as shown in Fig. 4c.^112^ The UV-visible absorption spectra in Fig. 4c1 showed that the copolymerization of g-C_3_N_4_ (CNU) with ATCN, ABN, DAMN, and BA induce a visible red-shift in all four copolymerized samples. Furthermore, a prominent absorption shoulder appears between 450 and 600 nm for all conjugated CNU samples, confirming enhanced visible-light harvesting. The shifting of photoluminescence (PL) spectra from shorter (460 nm for CNU) to longer wavelengths in CNU-ABN (530 nm) and CNU-ATCN (550 nm) is another evidence of reduction in band gap with extent and nature of copolymerization. From the PL spectra, it can be concluded that, the formation of surface dyadic heterostructures through copolymerization, greatly suppressed the charge recombination in g-C_3_N_4_ nanosheets by suppressing strong charge coupling phenomena.^123^

Enabling the n → π* electron T_2_ transitions by creating structural defects in g-C_3_N_4_ nanosheets is another promising approach (Fig. 4d) to extend the light absorption properties. As shown in Fig. 4d1, a remarkable red shift of the absorption edge to ∼600 nm was observed in highly polymerized g-CN-700 sample, compared to less polymerized g-C_3_N_4_ samples.^124^ By increasing the degree of polymerization, the structural distortions were induced in the tri-*s*-triazine units due to the breakage of hydrogen bonds present in the melon. This hydrogen-bond disruption at 700 °C produces a distinct absorption peak at ∼490 nm, corresponding to the n → π* electronic transition, which also deepens the material color from light yellow (pristine g-C_3_N_4_) to yellow–brown in g-CN-700. The co-polycondensation of urea and oxamide to form g-C_3_N_4_ nanotubes copolymer (CN-OA), clearly showed optical absorption above 465 nm, which is ascribed to the activation of n → π* electron transition. The color variation from pale yellow (pristine g-C_3_N_4_) to brownish yellow for OA-modified samples (CN-OA) further confirms the absorption of visible light beyond 450 nm as displayed Fig. 4d2 (inset). Notably, n → π* transitions are prohibited in perfectly symmetric planar structure, suggesting that OA-copolymerized g-C_3_N_4_ nanotube adopt an asymmetric planar structure which further facilitates charge-carrier transfer, thereby enhancing the photocatalytic activity.^125^

The deliberate creation of N-vacancies below π* (LUMO) orbitals, is another strategy to extend optical absorption, as a result low energy electronic transition (T_2_: π → n-vacancies) can be occurred more easily than the fundamental T_1_ transition (Fig. 4e). Post-synthesis thermal treatment of g-C_3_N_4_ in reducing environment (for example, hydrazine, NaBH_4_ and H_2_), often creates stoichiometric imbalance, increasing the C/N ratio due to the loss of N atoms which generates an unoccupied N-vacancy energy level below the π*. Consequently, these N-vacancies can red-shift the optical absorption of g-C_3_N_4_. For example, the g-C_3_N_4_ with N-vacancies (CN-*x*) has been synthesized by thermal polymerization of a mixture of urea and chemical reducing agent *i.e.* hydrazine (N_2_H_4_·H_2_O).^126^ The N_2_H_4_·H_2_O facilitated the formation of more reducing gases NH_3_ and H_2_, which cause the introduction of N-vacancies into the CN-*x* by eliminating N atoms from the C–N=C (N_2_C) sites within the triazine rings. These vacancies induce band-gap narrowing and create downward-shifted mid-gap states below the CB edge. Consequently, the visible-light absorption is extended, and the recombination of photogenerated electrons and holes is effectively suppressed. The UV-vis spectra (Fig. 4e1) showed a progressively red-shifted absorption edge in CN-*x* sample, compared to CN due to presence of N-vacancies. As depicted in Fig. 4e1 (inset), the color of CN-*x* gradually varies from yellowish-white to reddish-brown with increasing N_2_H_4_·H_2_O content. Moreover, a prominent Urbach tail (absorption tail) emerges in the visible-light region of CN-*x*, and the edge of this adsorption tail shifts toward higher wavelengths with increase in concentration of N-vacancies, which is generally responsible for forming the mid-gap states below π* (LUMO) orbitals (Fig. 4e2).^112^ Layered g-C_3_N_4_ can experience a homogeneous self-modification strategy to induce uniform N-vacancies, as interlayer spacing in g-C_3_N_4_ provide the path to diffuse the reducing agents (*e.g.*, hydrogen) from the surface to the bulk (Fig. 4e3). Moreover, hydrogen-bonded polymeric strands (*via* NH/NH_2_ groups) and weak interlayer van der Waals interactions in bulk melon, facilitate the creation of uniform N-vacancy upon hydrogen treatment at optimal temperature window of 520–540 °C without compromising the melon framework. The shifting of UV absorption spectra (Fig. 4e4), towards longer wavelength for reduced melon (R-melon) compared to pristine melon is attributed to the unform nitrogen vacancies. As result, the bandgap is reduced from 2.78 eV for pristine g-C_3_N_4_ (melon) to 2.03 eV for the reduced sample (R-melon).^127^

### 4.3 Synthesis of g-C_3_N_4_

The structural and electronic properties of g-C_3_N_4_-based photocatalysts strongly depend upon the synthesis parameters, such as choice of precursors, synthesis route, atmosphere, degree of polymerization, and nitrogen retention.^128^ Appropriate control of these parameters allows the optimization of surface area, porosity, crystallinity, and defect concentration, which are essential for effective charge separation and light absorption.

#### 4.3.1 Precursors

Synthesis of g-C_3_N_4_ involves a series of condensation reactions, and the pathway that is followed strongly depends upon the starting materials used.^129,130^ Precursors that are commonly used for synthesis of g-C_3_N_4_, such as cyanamide, dicyandiamide (DCDA), melamine, thiourea, urea, and others significantly differ in their chemical compositions.^130^ Therefore, even if synthesized under identical thermal conditions, the g-C_3_N_4_ obtained from different starting materials shows variation in structural, electronic and photocatalytic properties.^106^

Melamine is an extensively used precursor for synthesis of g-C_3_N_4_ because of its high N content and benign condensation pathway. Polymerization typically occurs around ∼550 °C to melon, with higher temperature (>∼600 °C) leading to decomposition, and the resulting material is relatively crystalline, with defined heptazine motifs (Fig. 5a).^27,36,131^ Cyanamide and DCDA have also been widely used for thermal polymerization of g-C_3_N_4_, offering efficient conversion while maintaining moderate structural order (Fig. 5b).^128,131^ Cyanamide provides molecular-level control of condensation, but its toxic nature poses handling issues.^131,132^ Urea is a low-cost and environment-friendly precursor, and is well suited for scalable thermal synthesis of g-C_3_N_4_.^129,131,133,134^ During thermal polymerization (Fig. 5c), it releases NH_3_/H_2_O/CO_2_, creating highly porous photocatalyst, however yields of urea-derived g-C_3_N_4_ are typically lower compared with melamine- or DCDA-derived g-C_3_N_4_. In contrast, thiourea introduces sulfur heteroatoms *via* self-doping in the polymeric framework of g-C_3_N_4_, due to *in situ* production of H_2_S production (Fig. 5d), which promotes visible-light absorption by narrowing the band gap (for example, from ∼2.7 eV to ∼2.63 eV).^131,135,136^

**Fig. 5:**
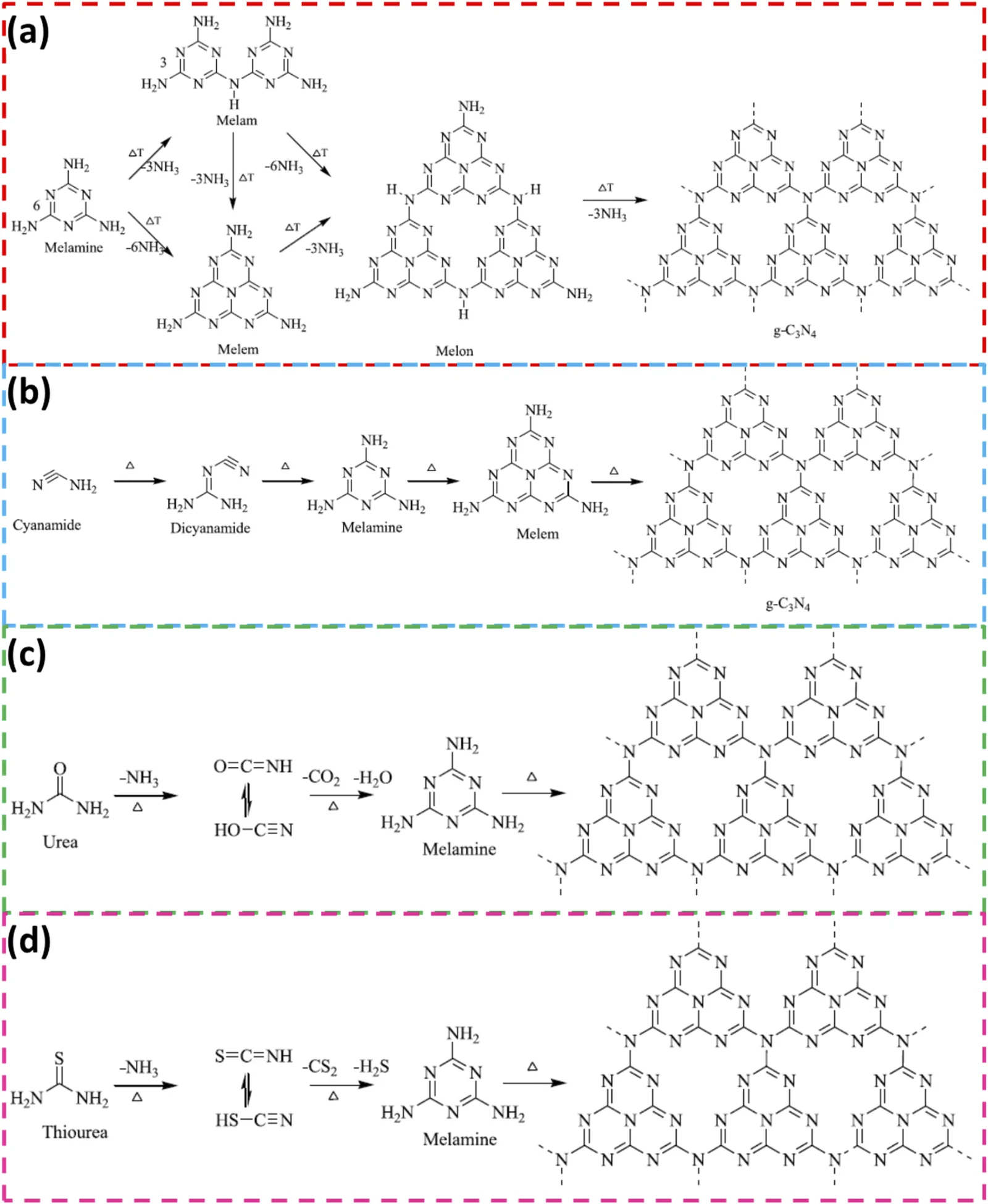
Stepwise schematic synthesis of g-C_3_N_4_ from (a) melamine, (b) cyanamide, dicyandiamide, (c) urea, and (d) thiourea, reproduced with permission from ref. 131 Copyright 2020 American Chemical Society.

In general, precursors with slower decomposition and higher nitrogen content yield more crystalline materials, while gas-releasing or dopant-rich sources produce higher porosity and more active sites.^129,137–139^ To compare the influence of different precursors, Table 3 summarizes representative synthesis conditions, C/N ratios, yields, surface areas, and corresponding structural characteristics of g-C_3_N_4_ obtained from commonly used N-rich precursors.

**Table 3:** Comparison of physicochemical characteristics of g-C_3_N_4_ synthesized from different nitrogen-rich precursors

Precursors	C/N ratio	Temperature (°C)	Yield (%)	Surface area (m^2^ g^−1^)	Structure	Ref.
Cyanamide	0.672	550	60–70	12	Partially crystalline	140
DCDA	0.69	600	53	26	Heptazine-based layered g-C_3_N_4_ with moderate crystallinity	128
Melamine	0.568	550	NR	12	Partially crystalline	141
Thiourea	0.695	600	11	36	Looser arranged layers	128
Urea	0.56	600	NR	54	Crystalline	142

#### 4.3.2 Synthesis techniques for g-C_3_N_4_-based catalysts

Numerous methods have been developed for synthesis of g-C_3_N_4_-based photocatalysts, which affects the degree of polymerization, porosity, surface area, and morphology. Following subsections will discuss some fundamental synthetic approaches and their effect on structural and electronic properties of obtained samples, which collectively influence their photocatalytic performance.

##### 4.3.2.1 Thermal polymerization

Thermal polymerization is the conventional and most reported method for the synthesis of g-C_3_N_4_-based catalysts. In a typical procedure, selected precursor is heated at 500–600 °C for 2–4 h in a muffle furnace under air or nitrogen atmosphere.^143^ It has been reported that higher temperatures enhance condensation of precursors into g-C_3_N_4_, while lower temperatures lead to incomplete polymerization.^144,145^ For instance, Yang *et al.* prepared g-C_3_N_4_*via* thermal polymerization and investigated the effect of temperature on the photocatalytic activity of g-C_3_N_4_.^146^ The catalyst CN-580-3-15, obtained by pyrolysis at 580 °C, exhibited better adsorption capacity (8.2 mg g^−1^ in 5 min), and achieved the highest methylene blue removal efficiency of 82%, compared to sample synthesized at low temperature.

The heating rate is also one of the important factors that influence the structure and properties of final product by affecting local ordering and defect density. For example, Yaemsunthorn *et al.* reported that the increase in heating rate during thermal polymerization of melamine, results in increased crystallite size, and surface area of obtained g-C_3_N_4_ catalysts.^147^

##### 4.3.2.2 Templated routes

Templated synthesis is another frequently used route to obtain highly porous g-C_3_N_4_, with increased surface area and greater number of active sites.^148,149^ Depending on the type of template, templating methods can further be categorized into hard templating, soft templating, and self-templating methods.^149–152^

In hard templating method the precursors are infiltrated into a rigid solid template, such as SiO_2_ nanoparticles, SBA-15, and KIT-6, which dictate the final morphology of g-C_3_N_4_. After calcination, the template is removed with the help of etching agents, such as HF, NaOH, or HCl.^153^ Hard templating method allows precise control over pore size, pore volume, and morphology.^154^ Moreover, hard templating methods are reported to synthesize mesoporous g-C_3_N_4_, with exceptionally high surface area, improved light utilization and enhanced photocatalytic activity. For example, Li *et al.* synthesized defect-rich porous g-C_3_N_4_ (dp-C_3_N_4_) photocatalyst, by using silica microsphere as hard template.^155^ The dp-C_3_N_4_ sample showed much higher BET surface area of 49.78 m^2^ g^−1^, compared to pristine g-C_3_N_4_ (15.60 m^2^ g^−1^). Moreover, the dp-C_3_N_4_ showed marked improvement in photodegradation efficiency towards RhB (88.83%), compared to g-C_3_N_4_ (18.7%), which can be correlated with higher accessible surface area and greater number of active sites.

On the other hand, soft templating method involves the use of self-assembling organic molecules or surfactant, such as Pluronic F127, P123, and CTAB. Self-assembly occurs between precursor and surfactant molecules upon their dissolution in the solvent (commonly water or ethanol).^154^ During calcination, organic templates decompose automatically, leaving behind highly porous g-C_3_N_4_, with large surface area.^156,157^ For example, Chen *et al.* reported the successful synthesis of porous g-C_3_N_4_ nanosheets *via* soft templating route using CTAC surfactant.^149^ The CN-1% CTAC sample showed increased surface area of 24.9 m^2^ g^−1^, compared to bulk g-C_3_N_4_ (14.4 m^2^ g^−1^), which was synthesized without using CTAC.

##### 4.3.2.3 Molten-salt routes

Molten-salt route has emerged as a promising strategy for large scale synthesis of highly crystalline, porous, and exfoliated g-C_3_N_4_ photocatalysts with high surface area and enhanced photocatalytic activity. In this method, nitrogen-rich precursors are mixed with molten inorganic salts, such as NaCl, KCl, LiCl, or their eutectic mixtures, followed by thermal polymerization.^158–160^ The salt uniformly melts around the precursor, forcing gases and volatile compounds to escape through the liquid, leaving behind a sponge-like network of micro-, meso-, and macro-pores.^161,162^ Dou *et al.* reported one-pot synthesis of Na-doped g-C_3_N_4_ (CN-0.05) *via* molten-salt route.^163^ The CN-0.05 catalyst achieved 99% photocatalytic degradation of RhB, which can be correlated with its high surface area (46.7 m^2^ g^−1^).

Molten-salt assisted synthesis can significantly improve the crystallinity and electronic structure of g-C_3_N_4_-based photocatalysts. For example, Shao *et al.* synthesized highly crystalline g-C_3_N_4_ with nitrogen vacancies, *via* a KCl/LiCl molten salt-assisted route, which resulted in improved crystallinity, tunable band structures, enhanced visible-light absorption, and better charge separation efficiency.^164^

##### 4.3.2.4 Microwave-assisted synthesis

Microwave-assisted synthesis of g-C_3_N_4_ is gaining a huge attention around the globe, particularly due to its ability to polymerize nitrogen-rich precursors at faster rate, with lower energy consumption compared to traditional thermal polymerization method.^165^ As microwave heating is caused by dipole rotation or ion migration, the precursors receive uniform heat during polymerization.^165,166^ Recently Said *et al.* synthesized g-C_3_N_4_/bauxite composite catalyst to study the effect of microwave-assisted synthesis route on the morphology and structural properties of composite.^167^ Scanning Electron Microscopy-Energy Dispersive X-ray Spectroscopy (SEM-EDS) revealed the formation of a highly porous microstructure, with homogeneous distribution of C, N, Al, Si, and O elements. In another study, Hoang *et al.* prepared Pd/g-C_3_N_4_ photocatalyst for degradation of SMX through a microwave-assisted method.^168^ The Pd/g-C_3_N_4_ catalyst showed excellent SMX removal efficiency of 97% compared to pristine g-C_3_N_4_, which achieved only 57% removal. The higher photocatalytic efficiency of Pd/g-C_3_N_4_ sample can be correlated with uniform dispersion of Pd nanoparticle over g-C_3_N_4_ nanosheets obtained *via* microwave-assisted synthesis, which improved interfacial charge transfer and suppressed recombination.

##### 4.3.2.5 Chemical vapor deposition (CVD)

The CVD is a bottom-up technique, frequently used to deposit homogenous film of g-C_3_N_4_ onto a substrate, such as quartz glass and silicon wafers.^122,169–172^ In this method, nitrogen-rich precursors are thermally decomposed in a heated chamber, followed by surface-assisted polymerization and deposition of highly crystalline g-C_3_N_4_ onto the substrate.^122,173^ Furthermore, CVD allows the growth of ultrathin, 2-D g-C_3_N_4_ films without requiring post-synthesis exfoliation.^122^ For example, Wu *et al.* demonstrated the successful synthesis of g-C_3_N_4_ nanosheets with larger crystalline domains (up to 40 µm), from melamine precursor *via* CVD approach, using Cu foil as a substrate.^174^ XPS studies showed the absence of amino-related peaks, which indicated nearly complete polymerization of melamine molecules to highly crystalline g-C_3_N_4_. The optical microscopy analysis showed parallel triangular flakes with smooth and clear edges, suggesting the epitaxial growth of flakes with single crystal orientation. EDS analysis further confirmed the homogenous distribution of carbon and nitrogen atoms. These findings highlight the feasibility of CVD for large scale production of continuous g-C_3_N_4_ films for practical applications.

##### 4.3.2.6 Emerging synthetic approaches

From past few years, AI-assisted optimization of synthesis parameters is gaining huge interest of researchers.^175–177^ Machine learning and AI-driven models can predict the most suitable synthesis conditions, such as precursors ratios, heating temperature, heating rate, and reaction time. For example, Yurova *et al.* used machine learning models to optimize the calcination temperature, heating rate, and reaction time for synthesis of g-C_3_N_4_ photocatalysts.^177^ The developed model successfully identified the suitable synthesis parameter, with high prediction accuracy of *R*^2^ > 0.9.

Supramolecular assembly is another emerging approach for synthesizing highly porous g-C_3_N_4_-based photocatalysts.^178–180^ In this method, the solution of nitrogen-rich precursors is stirred or sonicated to initiate assembly, often followed by hydrothermal treatment. The solid supramolecular complex is then subjected to thermal polymerization to form final g-C_3_N_4_ catalyst.^181–185^ Bai *et al.* demonstrated the successful synthesis of 3-D porous g-C_3_N_4_ photocatalyst, using melamine and oxalamide *via* supramolecular self-assembly strategy.^179^ SEM and TEM analysis confirmed the formation of sponge-like porous g-C_3_N_4_ structure, with improved surface area of 59.41 m^2^ g^−1^, compared with bulk g-C_3_N_4_ (9.65 m^2^ g^−1^). The prepared sample showed an excellent photodegradation efficiency of 88.8% toward degradation of TC, which can be linked to its high surface area and narrowed bandgap (2.02 eV).

Plasma-assisted synthesis is another emerging technique for preparation of vacancy-rich and surface functionalized g-C_3_N_4_-based photocatalysts, requiring comparably low temperature.^186–188^ This method provides the precise control over surface functionalities, defect concentrations and particle sizes, resulting in excellent photocatalytic activity of samples.^189–192^ Hao *et al.* reported the successful synthesis of ZnO/g-C_3_N_4_ heterojunction for degradation of deoxynivalenol (DON), *via* plasma-assisted hydrothermal method.^186^ The XPS and DFT studies revealed the presence of plasma-assisted oxygen vacancies in the samples, which can act as electron trapping sites to suppress charge recombination. The optimized sample showed 100% DON degradation within 90 min, which was 3.78 times higher than that of pristine ZnO. Table 4 presents a comparative overview of various synthesis approaches used for the preparation of g-C_3_N_4_-based photocatalysts, along with their effects on the structural and physicochemical properties of the resulting materials.

**Table 4:** Comparison of major synthesis routes for g-C_3_N_4_, highlighting typical precursors, processing conditions, surface area, resulting structural features, advantages, and limitations, as reported in representative studies

Synthesis approach	Precursors	Conditions	Structural features	Advantages	Limitations	Ref.
Thermal polymerization	Urea, melamine	550 °C, 3 h	Bulk layered structure	Simple, low-cost, scalable, metal-free route	High energy consumption, possible agglomeration, charge recombination in bulk structures	193
Hard-templating route	Cyanamide, SiO_2_ microspheres, HF (etchant)	550 °C, 4 h	Porous g-C_3_N_4_ with honeycomb-like mesoporous structure	Improved visible-light absorption, suppressed charge carrier recombination, improved charge transfer kinetics	Requires hazardous etchants for template removal, multi-step high-temperature synthesis, photocatalytic efficiency decreases after repeated cycles	155
Soft-templating route	Melamine, CTAC	550 °C, 4 h	Porous, ultrathin nanosheets, with curly and wrinkled morphology	Environment-friendly, low-cost, no need of harmful etchants	Photocatalytic enhancement strongly depends upon the optimal soft template loading, excessive loading can decrease activity, moderate surface area compared with hard-templating methods	149
Molten-salt route	Melamine, NaCl aqueous solution (as molten-salt precursor modifier)	550 °C, 4 h	Crystalline and mesoporous g-C_3_N_4_ with enhanced interlayer ordering, increased surface area, and narrowed bandgap	Simple and scalable synthesis, improved crystallinity, improved charge separation	Excessive molten salt can cause over-crystallization and structural collapse, residual ion may affect purity, morphology control is less precise compared to hard-templating or CVD	194
Microwave heating	Urea	Optimum microwave power: 800 W, 15 min	Porous honeycomb-like g-C_3_N_4_ nanosheets with interconnected mesopores	Ultrafast synthesis, lower energy consumption, template-free process	Limited morphology control compared with templating methods possible microwave hot-spot formation	195
CVD	Melamine	Source temperature: 400 °C, substrate temperature: 600 °C, growth time: 30 min	Crystalline g-C_3_N_4_ thin films, monolithic growth on crystalline and non-crystalline substrates	Direct vapor-phase growth of crystalline g-C_3_N_4_ film, excellent uniformity, controlled film growth and thickness	Requires specialized CVD instrumentation, high temperature requirement, possible thermal degradation and surface contamination	196

## 5 Characterization and kinetic modeling of g-C_3_N_4_ based photocatalysts

With the recent growing interest in the field of g-C_3_N_4_-based for CECs degradation, the development of advanced characterization techniques for such systems also becomes very important. These techniques not only help in understanding the structure–property relationship of catalysts, but also provide useful mechanistic insights. This section summarizes the recent advancements in physicochemical, kinetic and electrochemical methodologies used to evaluate the performance of g-C_3_N_4_-based photocatalysts for contaminant degradation.

### 5.1 Physicochemical characterization of g-C_3_N_4_

The structural and compositional analysis of g-C_3_N_4_ is very important for understanding its physicochemical properties and photocatalytic performance. The tri-*s*-triazine units connected by planar amino groups generally form the graphitic planes in g-C_3_N_4_. X-ray diffraction (XRD) provides the fundamental evidence of phase formation and interlayer stacking within the tri-*s*-triazine framework. As shown in Fig. 6a, broad diffraction features around 13° (*d*_1_ ≈ 0.713 nm) and 27° (*d*_2_ ≈ 0 : 326 nm) correspond to in-plane structural motifs and graphitic layer ordering, while the change in peak intensity after modification indicates the altered crystallinity or interlayer spacing.^36,197^

**Fig. 6:**
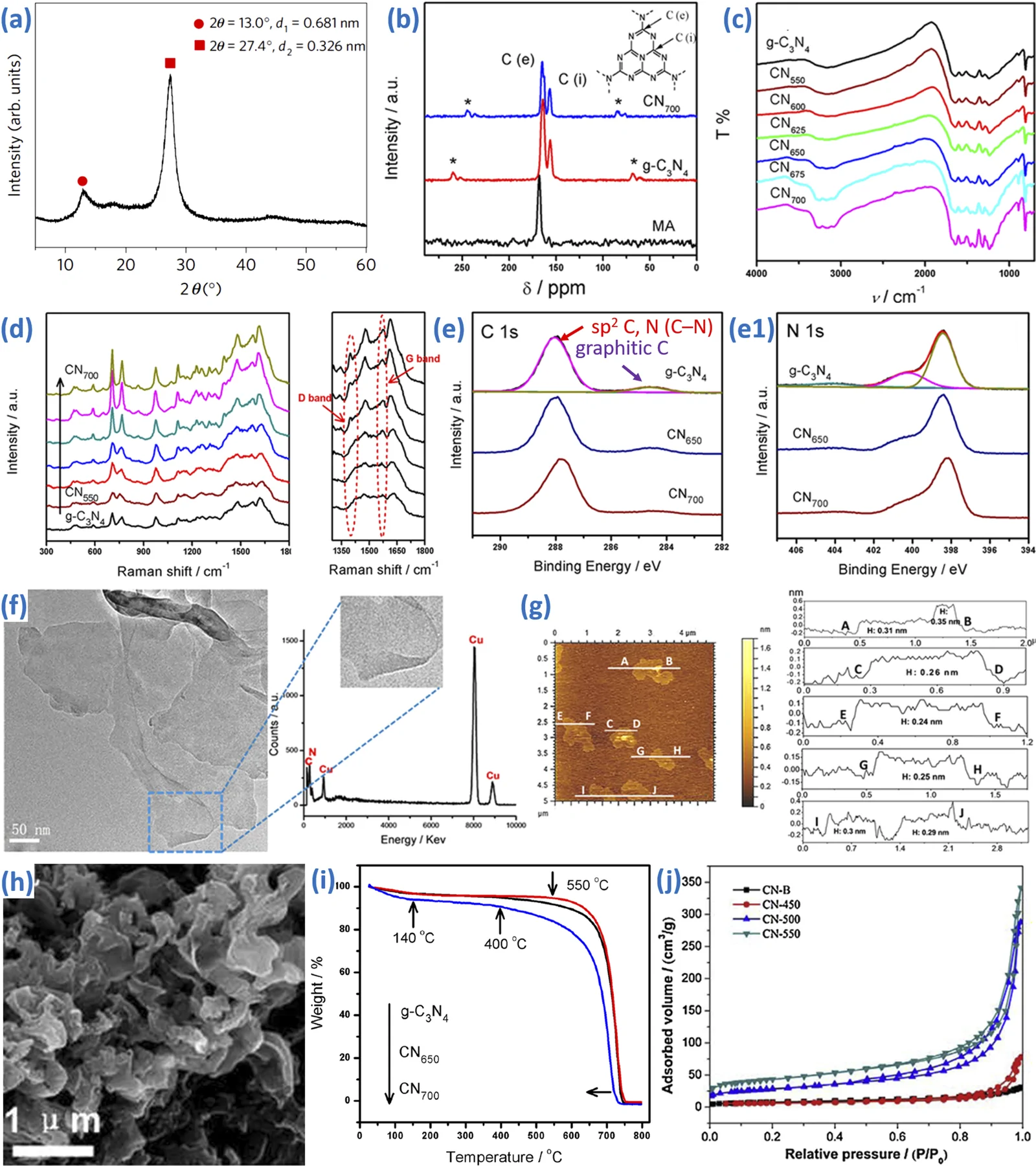
(a) XRD pattern of the g-C_3_N_4_, confirming a graphitic structure with an interplanar stacking distance of 0.326 nm, adopted from open source ref. 204 Copyright 2022 MDPI (Basel, Switzerland); comparison of (b) solid-state ^13^C NMR, (c) FTIR, (d) Raman spectra, high-resolution XPS spectra of (e) C, (e1) N for g-C_3_N_4_ (CN-*x*) samples polymerized at different temperature; (f) TEM and corresponding EDS spectra. (g) AFM image and height profiles of CN-700 nanosheets, reproduced with permission from ref. 114 Copyright 2014 American Chemical Society; (h) SEM image of g-C_3_N_4_, reproduced with permission from ref. 123 Copyright 2014 Royal Society of Chemistry; (i) comparison of TGA curves of different CN samples under N_2_ atmosphere (heating rate:10 K min^−1^), reproduced with permission from ref. 114 Copyright 2014 American Chemical Society; and (j) N_2_ adsorption–desorption isotherms g-C_3_N_4_ samples polymerized at different temperature, reproduced with permission from ref. 203 Copyright 2015 Elsevier B.V.

Furthermore, the solid-state ^13^C NMR, FTIR, Raman spectroscopy, and XPS are frequently used to determine the chemical structure and composition of the g-C_3_N_4_. Two prominent peaks at ∼156–158 and ∼162–165 ppm (Fig. 6b), compared to single sharp line of melamine (MA), in NMR spectra, are assigned to C atoms, that are directly attached to the bridging N (C_1_–N_bridge_) and the N atoms (C_2_–N_amine_) connecting the rings within the tri-*s*-triazine (heptazine) units, respectively.^114,198^ FTIR spectroscopy reveals stretching and bending vibrations of C–N–C linkages, enabling identification of heteroatom doping and defect sites.^199^ The bands at approximately 1550–1640, 1200–1400, and 802 cm^−1^ (Fig. 6c) are the characteristics stretching vibration mode of –C=N, –C–N/and breathing tri-*s*-triazine units, respectively.^112,114^ Raman analysis complements FTIR by probing lattice disorder and π-conjugation within the aromatic domains. As displayed in Fig. 6d, the 1200–1700 cm^−1^ region corresponds to stretching modes of tri-*s*-triazine ring, while the peaks at ∼705 and ∼980 cm^−1^ are attributed to C–N–C bending and breathing vibrations of heptazine or *s*-triazine units. Raman peaks at 1405 and 1570 cm^−1^ are characteristic spectrum of graphene and layered C–N materials, the higher D band to G band ratio (*I*_D_/*I*_G_) indicates the structural distortion or defect-induced sp^2^ carbon sites.^112,114^ XPS quantifies elemental states, identifying chemical bonding environments and electronic shifts related to doping or defect formation.^200,201^ In the high-resolution XPS spectra of C 1s for g-C_3_N_4_, a main peak at 288.0 eV corresponds to sp^2^ C atom attached with N (C–N) in aromatic rings, and a smaller peak at 284.6 eV corresponds to graphitic C (Fig. 6e). In the deconvoluted N 1s spectrum (Fig. 6e1), the peaks at 398.4, 400.3, and 404.1 eV are assigned to sp^2^ N atoms in triazine rings, bridging N in N–(C)_3_, and charging effects, respectively.^114^

The transmission electron microscopy (TEM) and atomic force microscopy (AFM) clarify the nanosheet thickness, particle dispersion, and interface contact in g-C_3_N_4_ composite systems. The combination of these techniques illustrates whether dopants or secondary phases are homogeneously integrated into the g-C_3_N_4_ matrix.^202^ The thin, glossy, and transparent atomic layers of 2-D g-C_3_N_4,_ stabilized by a distinct curvature at the edge of plane are shown in TEM image along with EDS analysis (Fig. 6f). The height of the boundary and interior planes of g-C_3_N_4_ nanosheets found to be in the range of 0.2–0.4 nm as revealed in AFM analysis (Fig. 6g).^114^ The surface morphology and modified texture of g-C_3_N_4_ are investigated by scanning electron microscopy (SEM), the SEM image (Fig. 6h) revealed a stacked, agglomerated particle and sheets type morphology of g-C_3_N_4_.

The thermogravimetric analysis (TGA) is widely used to determine the thermal stability of g-C_3_N_4_ samples. The Fig. 6i revealed that the samples polymerized at 550 and 600 °C exhibited better stability compared to prepared at 700 °C. This slight decrease in stability may be attributed to changes in local packing associated with reduced dimensionality.

Brunauer–Emmett–Teller (BET) surface analysis and pore architecture, key descriptors of adsorption and active-site density. The BET surface area of g-C_3_N_4_ increased from 4.0 m^2^ g^−1^ to 99 m^2^ g^−1^, with the increase in degree of polymerization at elevated temperatures.^114^ Thermal exfoliation of bulk g-C_3_N_4_ (CN-B) at temperature 550 °C for 2 h in air, has also been reported to improve the surface area up to 151 m^2^ g^−1^ as shown in Fig. 6j.^203^

### 5.2 Photocatalytic activity evaluation and kinetics

Optical properties of g-C_3_N_4_-based photocatalysts are typically assessed by diffuse reflectance spectroscopy (DRS), from which band-gap energies are estimated using Tauc relationships. A red-shift in absorption onset usually indicates band modulation through heteroatom substitution or interface coupling. Fig. 7a shows DRS of samples prepared by thermal polymerization. Samples synthesized up to 625 °C exhibit a single absorption edge around 450 nm, corresponding to the intrinsic π → π* transitions of g-C_3_N_4_. With increasing polymerization temperature from 550 to 625 °C, a slight red shift of the absorption edge is observed due to enhanced π-electron delocalization. However, a noticeable blue shift is observed above 650 °C, attributed to quantum confinement from thermally induced size reduction. Moreover, a new absorption edge (∼500 nm) appears above 650 °C, likely due to enhanced n → π* transitions during thermal exfoliation, improving visible-light absorption.^114^

**Fig. 7:**
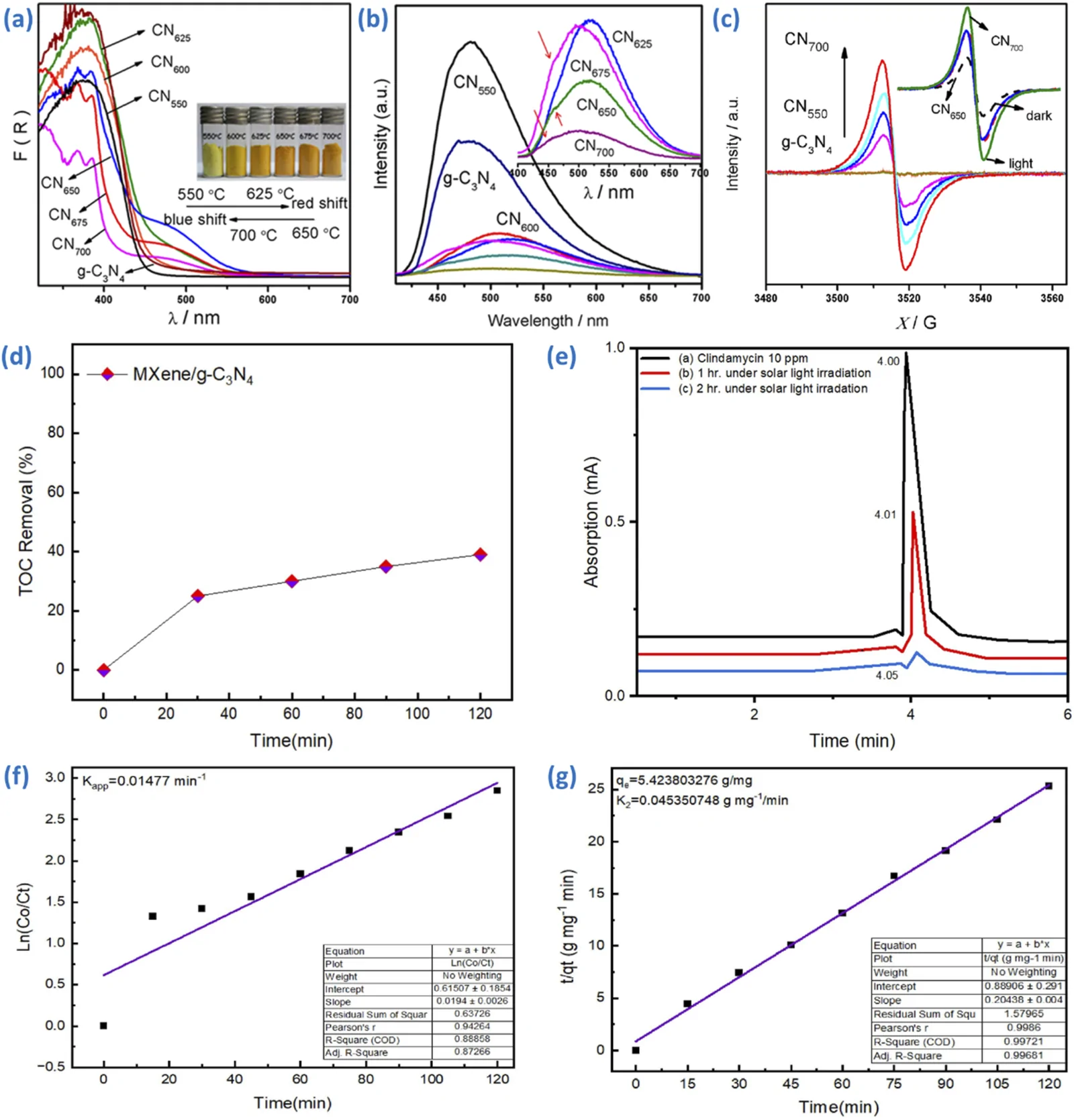
Evaluation of photocatalytic activity for g-C_3_N_4_-based systems (a) optical-absorption, (b) fluorescence emission, (c) room-temperature EPR spectra, reproduced with permission from ref. 114 Copyright 2014 American Chemical Society; (d) photocatalytic total organic carbon (TOC) removal over time for MXene/g-C_3_N_4_ composite; (e) HPLC chromatograms showing clindamycin degradation under solar light; clindamycin removal kinetics (f) pseudo-first-order and (g) pseudo-second-order, reproduced with permission from ref. 215 Copyright 2025 Elsevier B.V.

Photoluminescence (PL) and time-resolved PL (TRPL) measurements are widely used to monitor charge-carrier recombination dynamics. The decrease in PL intensity is generally correlated with improved electron–hole separation.^205,206^ A similar red shift (below 650 °C) and a blue shift (above 650 °C), in the range of 450–510 nm are observed in PL spectra (Fig. 7b), that is in accordance with DRS spectra. This emerges from quantum size confinement in nanoscale carbon nitride polymers with reduced dimensions, caused by thermal decomposition.^207^ The PL intensity decreases at higher degree of polymerization due to extension of the π-conjugated system, that enhance the π states and orbital overlap. The PL intensity is further reduced by rapid charge transfer at buckled sites, which improve photocatalytic reactions.^114,208^ Room-temperature electron paramagnetic resonance (EPR) spectroscopic analysis is a powerful technique to determine the unpaired electrons in the π conjugated aromatic rings, paramagnetic defects, free radicals and transition metal ions which are often used to evaluate the electronic band structure, coordination geometry and oxidations states of semiconductor materials.^209,210^ As displayed in Fig. 7c, the increased Lorentzian line intensity in the dark revealed the temperature-driven amplification of the π conjugation without disturbing its original conjugated system. The inset displays a further increased EPR signal under visible-light irradiation, confirming efficient photochemical generation of radical pairs in highly polymerized g-C_3_N_4_ semiconductor. Collectively, all these analyses form the basis for linking material structure to photocatalytic function.^114^

Moreover, the accurate evaluation of photocatalytic performance requires well-defined experimental conditions and quantitative kinetic modeling. Pollutant photodegradation studies are usually conducted under sunlight or visible light irradiation, using aqueous solutions of targeted contaminants. The changes in pollutant concentration are usually monitored by high-performance liquid chromatography (HPLC) or liquid chromatography-mass spectrometry (LC-MS), while total mineralization is measured by total organic carbon (TOC) or chemical oxygen demand (COD).^211–213^ As shown in Fig. 7d, the clindamycin is effectively removed up to 38% within 120 min, from natural wastewater using the magnetic g-C_3_N_4_/MXene. The significant reduction in HPLC peak intensity after 1 and 2 h of solar irradiation indicates effective photodegradation of pharmaceutical clindamycin, using g-C_3_N_4_-based photocatalyst (Fig. 7e).

Kinetic interpretation frequently follows the Langmuir–Hinshelwood (L–H) pseudo-first-order model (eqn (9)), which describes the surface reaction between adsorbed species and photogenerated oxidants.^214^ In adsorption-assisted photocatalytic systems, pseudo-second-order kinetic models (eqn (10)), is also sometimes employed to evaluate adsorption-controlled interfacial processes.9
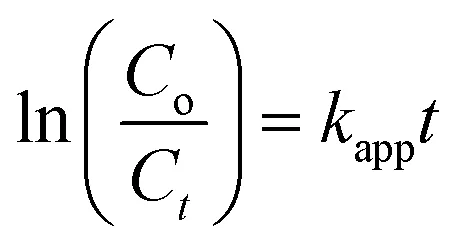
10
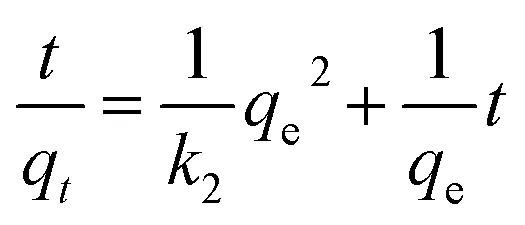
In eqn (9) and (10), *k*_app_ and *k*_2_ represents the apparent first order rate constant (min^−1^) and the second order rate constant (g mg^−1^ min^−1^), respectively. The *C*_o_ and *C*_*t*_ are the initial and instantaneous pollutant concentrations. While *q*_*t*_ (mg g^−1^) and *q*_e_ (mg g^−1^) represents the photodegraded amount of pollutant at time *t* (min) and degradation capacity at equilibrium. Reporting *k*_app_, *k*_2_ and degradation percentage under clearly defined illumination, catalyst dose and pH are essential for meaningful comparison between studies. The magnitude of these kinetic constants provides insight into the degradation efficiency and surface reaction behavior of photocatalytic systems. Higher *k*_app_ and *k*_2_ values generally indicate faster degradation kinetics and more efficient pollutant removal. For example, as shown in Fig. 7f and g, the evaluated *k*_app_, *k*_2_ and *q*_e_ values were 0.01477 min^−1^, 0.04535 g mg^−1^ min^−1^ and 5.4238 g mg^−1^, respectively, demonstrating efficient adsorption-assisted photocatalytic activity of MXene/g-C_3_N_4_ towards clindamycin.^215^

### 5.3 Electrochemical and spectroscopic mechanistic tools

Electrochemical impedance spectroscopy (EIS) is frequently used technique to investigate the charge transfer behavior, interfacial electron transport kinetics, and conductivity of g-C_3_N_4_-based photocatalysts.^216–219^ A small semicircle in Nyquist plots indicates lower resistance and more efficient separation of electron and h^+^, while smaller *R*_ct_ values usually correspond to improved conductivity, better electronic coupling, and accelerated electrocatalytic reactions.^220–223^ In addition to EIS, surface photovoltage (SPV) analysis is also frequently used to investigate the separation efficiency of photogenerated charge carriers.

For example, Zhao *et al.* prepared a BiPO_4_/g-C_3_N_4_-B Z-type heterojunction *via* co-precipitation method, for photocatalytic degradation of MB.^224^ As shown in Fig. 8a, pristine BiPO_4_ exhibited a relatively large Nyquist semicircle, indicating higher interfacial charge-transfer resistance and slower carrier migration. In contrast, the B-doped g-C_3_N_4_ nanosheets (g-C_3_N_4_-B) showed improved conductivity due to enhanced π-conjugation after B incorporation. Among all investigated samples, the 2-D/2-D BiPO_4_/g-C_3_N_4_-B heterojunction with a 1 : 2 mass ratio (1 : 2 BP-CB) showed the smallest Nyquist radius, confirming the lowest interfacial resistance and the most efficient photogenerated charge separation after heterojunction formation. Furthermore, SPV spectroscopy (Fig. 8b) showed that both g-C_3_N_4_-B and 1 : 2 BP-CB generated stronger surface photovoltage signals compared to pristine BiPO_4_, indicating more efficient light-induced separation of electron–hole pairs. The excellent photocatalytic activity of 1 : 2 BP-CB sample (97.3%) can be correlated with improved charge separation after heterojunction formation.

**Fig. 8:**
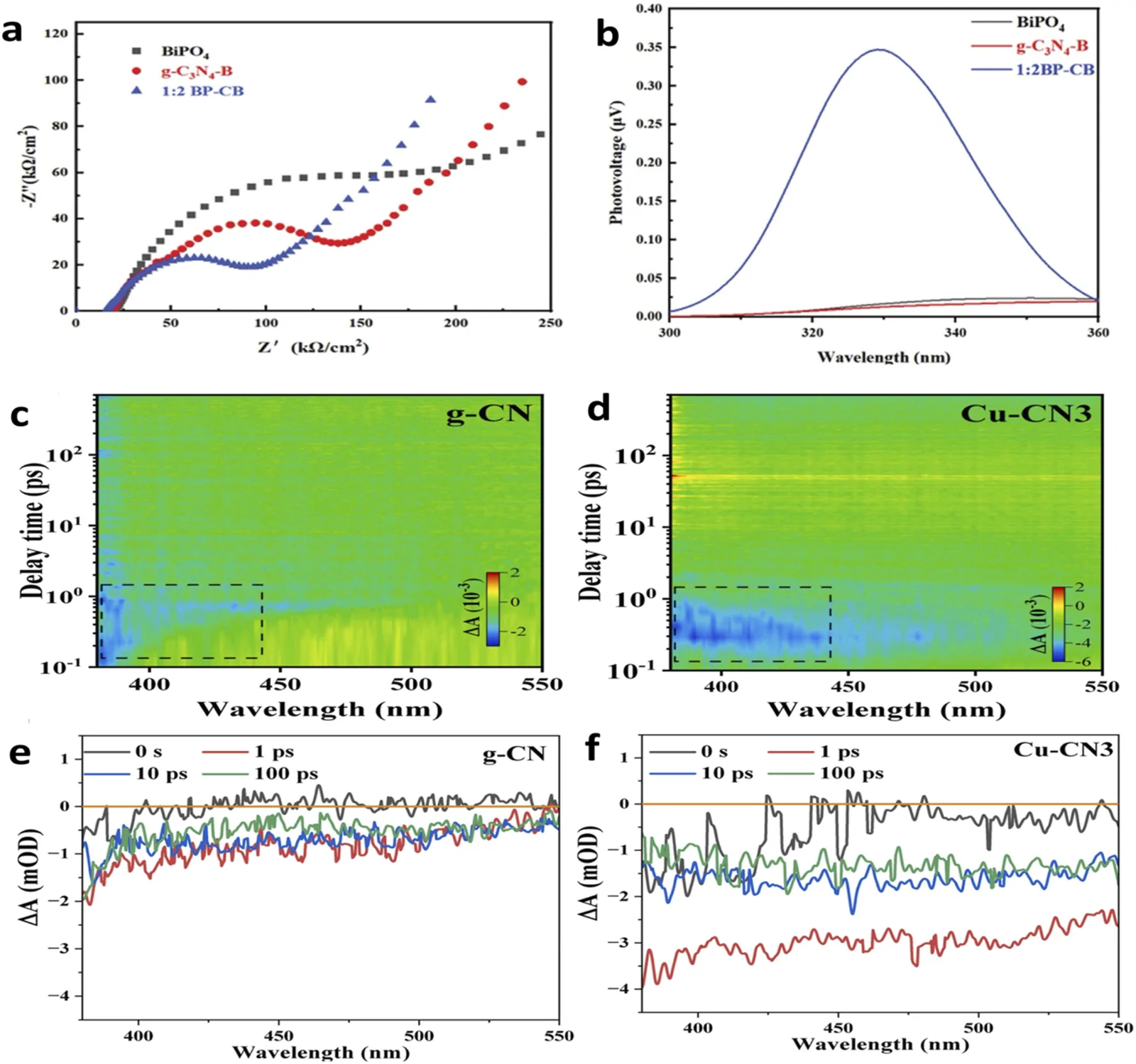
Electrochemical and ultrafast spectroscopic characterization of modified g-C_3_N_4_-based photocatalysts (a) EIS Nyquist plots and (b) SPV spectra of BiPO_4_, g-C_3_N_4_-B, and 1 : 2 BP-CB heterojunction systems; reproduced with permission from ref. 224 Copyright 2023 Springer Nature; (c and d) fs-TA spectra and (e and f) corresponding transition absorption spectra of pristine g-C_3_N_4_ (g-CN) and Cu-MOF/g-C_3_N_4_ (Cu-CN3), respectively, reproduced with permission from ref. 225 Copyright 2025 Elsevier B.V.

Transient absorption spectroscopy (TAS) has emerged as a powerful tool to track carrier lifetimes on the femtoseconds (fs) to µs scale, especially in Z and S-schemes heterostructure. For example, Sabir *et al.* performed fs-TAS to investigate charge carrier dynamics in Cu-MOF/g-C_3_N_4_ (Cu-CN3), a S-scheme photocatalyst.^225^ As shown in fs-TAS plots (Fig. 8c–f), the Cu-CN3 heterojunction showed stronger and intense absorbance difference (Δ*A*) signal over a wider spectral range, *i.e.* 380–500 nm, due to extended absorption of Cu-MOF, compared to pristine g-C_3_N_4_. Moreover, the Cu-MOF/g-C_3_N_4_ heterojunction showed an ultrashort lifetime of 4.7 ps, suggesting ultrafast interfacial electron transfer across the heterointerface.

## 6 Modification strategies for g-C_3_N_4_-based photocatalysts

As previously discussed, pristine g-C_3_N_4_ suffers from some inherent limitations, such as low quantum yield, limited visible-light response, fast charge recombination, and limited surface area. Over the past decade, many strategies have been employed to overcome these limitations and improve the photocatalytic activity of g-C_3_N_4_. These strategies (Fig. 9a), include doping, co-doping, heterojunction formation, carbon-based hybrids, morphology engineering, and porosity control.^226–231^ This section will review these strategies with special focus on their mechanistic implications, structural impact (Fig. 9b), and their influence on the photocatalytic performances of g-C_3_N_4_ for CECs degradation.

**Fig. 9:**
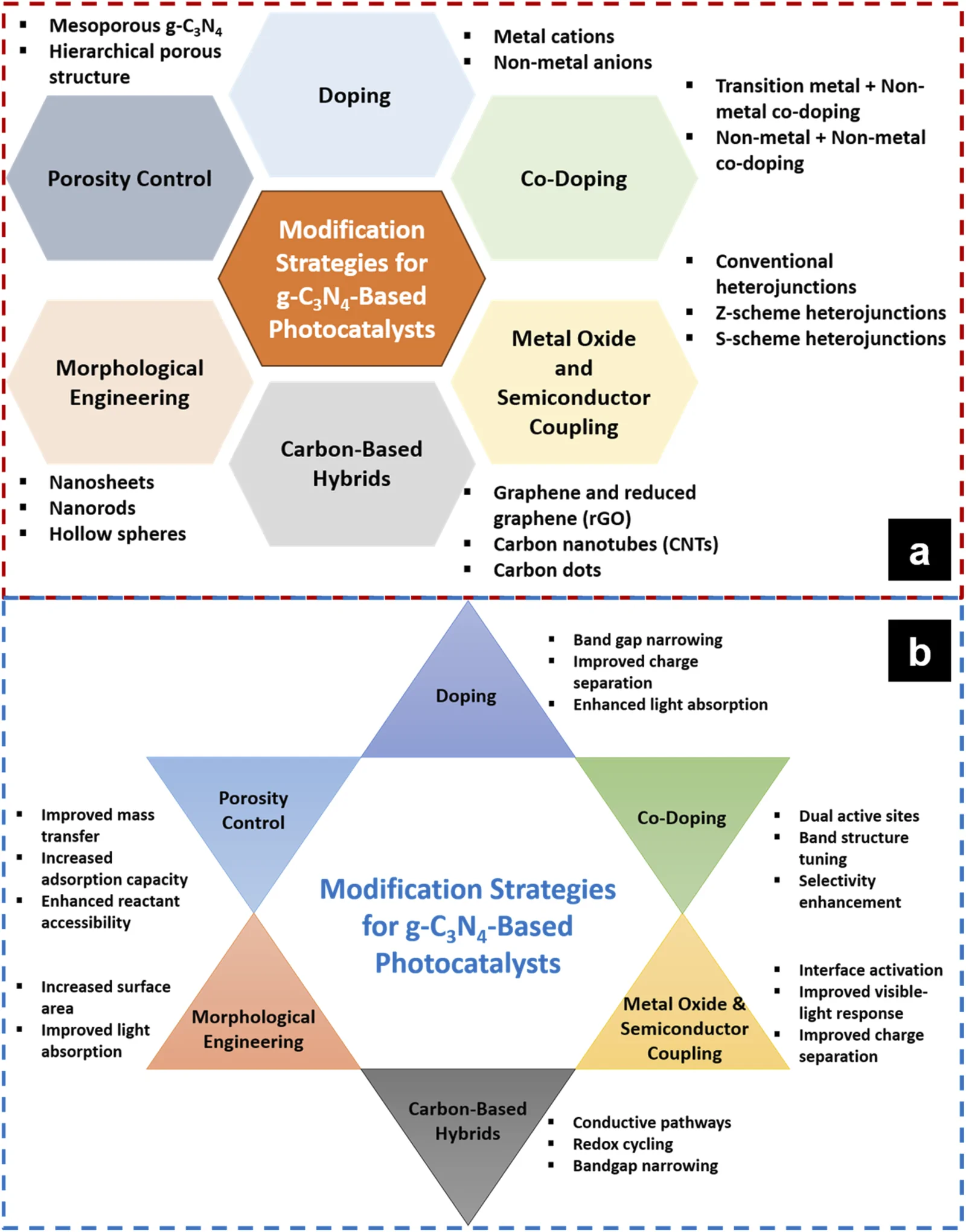
(a) Schematic overview of key modification strategies for g-C_3_N_4_-based photocatalysts and (b) impact of various modification approaches on the photocatalytic performance of g-C_3_N_4_-based materials.

### 6.1 Doping

Doping pristine g-C_3_N_4_ is an effective modification technique, which is frequently used to tune the bandgap, improve UV-visible light absorption and suppress charge carrier recombination.^232–234^ Depending on the type of dopant, doping can be classified into metal and non-metal doping.

Metal doping of g-C_3_N_4_ has emerged as a powerful strategy to improve its photocatalytic activity for CECs removal. Introduction of metal dopant creates mid-gap energy level near the CB or VB, which can act as electron trap site, hence improving the charge-carrier separation and electrical conductivity.^235–237^ Transition metal doping, in particular is reported to improves the visible light absorption and facilitates charge carrier separation during photocatalytic reactions.^236^ Lu *et al.* reported the successful synthesis of Fe-doped g-C_3_N_4_*via* ultrasonic route.^238^ The prepared photocatalyst showed 2.1- and 2.6-times higher degradation rates for MO and TC, respectively, relative to pristine g-C_3_N_4_, which can be correlated with broadened light absorption, accelerated charge separation, and O_2_˙^−^ radical dominated pathways. Incorporation of Co into g-C_3_N_4_*via* thermal polymerization generated intercalation sites that enhanced visible-light absorption and established efficient electron transfer channels, enabling CoCN_0.02_ to achieve 97% TC degradation within 30 min.^239^ Ba doping into g-C_3_N_4_*via* thermal condensation narrowed the band gap, increased surface area, and suppressed electron–hole recombination, enabling 91.9% TC degradation under visible light.^240^ Introducing Ag into the g-C_3_N_4_ lattice broadened its optical response and facilitated rapid charge migration, allowing the optimized 0.1-AgCN photocatalyst to achieve 84% CIP removal (over twice that of pristine g-C_3_N_4_).^241^ Atomically dispersed Mo anchored onto g-C_3_N_4_ nanosheets tuned the electronic structure, increased the surface area and formed an amorphous–crystalline interface, enabling Mo/CN-0.075 to achieve 92% tetracycline removal through efficient charge separation.^242^

Non-metal dopants such as B, P, S, and O typically substitute the carbon or nitrogen atoms present in the heptazine ring and modulate the electronic structure by creating defect sites, which results in narrowing of bandgaps and improved charge transfer.^243,244^ In a representative study, incorporation of B into the g-C_3_N_4_ effectively narrowed its band gap, improved visible-light absorption, and promoted charge separation, enabling a 5.2-fold increase in the photodegradation rate of SMX compared to pristine g-C_3_N_4_ under visible irradiation.^245^ Similarly, P incorporation into the g-C_3_N_4_ framework reduces its band gap from 2.70 to 2.48 eV and enhances the concentration of electron–hole pairs, improving the photodegradation of SMX and CIP, achieving up to 70% and 60% removal, respectively.^246^ O doping into the g-C_3_N_4_ perturbed its electronic symmetry and facilitated charge separation, while promoting the generation of O_2_˙^−^, as a result, OCN-1.5 achieved 82.6% BPA removal under visible light, which is approximately 60% improvement compared to pristine g-C_3_N_4_.^247^ Zhang *et al.* (2022) prepared O-doped porous g-C_3_N_4_ (CNUC) from urea and ammonium oxalate, achieving a tuned electronic structure that promoted efficient charge separation and generation of O_2_˙^−^ and h^+^, resulting in a 7-fold enhancement in BPA degradation under visible light as compared to pristine g-C_3_N_4_.^248^ Semi-ionic F-doping into g-C_3_N_4_ narrowed the bandgap to 2.55 eV and strengthened charge separation through polar C–F bonds, enabling GCN-F0.50 to achieve 99.8% TC removal under visible light (*λ* > 420 nm), a marked improvement over pristine g-C_3_N_4_.^249^ Moreover, carbon self-doping of g-C_3_N_4_ achieved through a one-pot urea–ammonium citrate route, introduced C–O–C linkages that narrowed the band gap and improved electron–hole separation, enabling C_0.02_CN to achieve 96% BPA removal under visible light (6.7 times higher than pristine g-C_3_N_4_).^250^ In another study, I-doping, accompanied by carbon-vacancy formation, modulated the electronic structure of g-C_3_N_4_ by generating electron-rich defect sites and narrowing the band gap to 2.45 eV, thereby extending visible-light absorption and minimizing charge recombination, which yielded a 2.6-fold enhancement in TC degradation relative to pristine g-C_3_N_4_.^251^ The I-doped g-C_3_N_4_ sample show significantly higher photodegradation efficiency and faster apparent rate constants compared to undoped g-C_3_N_4_. The radical scavenger experiments further confirmed the O_2_˙^−^ and h^+^ dominated oxidation pathway, and improved charge separation due to I doping and vacancy formation.

### 6.2 Co-doping

Metal and non-metal co-doping has emerged as a powerful approach to synergistically tune the band structure, enhance light harvesting, and improve interfacial charge separation in g-C_3_N_4_-based photocatalysts. Unlike mono-doping, which often provides limited enhancement or induces trap states, co-doping creates complex defect states that balance redox potentials, reduce recombination, and extend photo-response into longer wavelengths.^252–254^ This dual-doping approach also facilitates localized polarization, forming internal electric fields that drive directional charge migration crucial for effective CEC degradation.^255,256^

In a representative study Ni, S co-doped g-C_3_N_4_ was synthesized *via in situ* growth strategy, which provided abundant electron-rich sites and increased the available surface area.^160^ The resulting photocatalyst achieved a TC degradation rate of 0.032 min^−1^, 6 times higher than the pristine g-C_3_N_4_. Xu *et al.* introduced P and Mo simultaneously into g-C_3_N_4_ through a simple thermal treatment, producing nanosheets with abundant cyano groups and nitrogen vacancies, an enlarged surface area, and improved absorption of visible light.^254^ This co-doped material displayed a TC degradation rate of 0.01 min^−1^, which is 3 times greater than that of pristine g-C_3_N_4_, due to the efficient charge separation and O_2_˙^−^ and h^+^ dominated degradation pathway. Similarly, simultaneous co-doping of Fe and P into g-C_3_N_4_ suppressed crystal growth, increased the surface area, narrowed the band gap, and reduced charge-carrier recombination, resulting in markedly improved rhodamine B degradation as compared to pristine or mono-doped g-C_3_N_4_.^257^ A catalyst composed of atomically dispersed Cu on oxygen-enriched g-C_3_N_4_ nanosheets, prepared by pre-assembly calcination, displayed outstanding ability to activate peroxymonosulfate (PMS) under visible light, producing abundant ^1^O_2_ and enabling rapid CBZ degradation through the combined effects of photocatalysis and PMS activation.^258^

Incorporating two metals into the g-C_3_N_4_ framework provide a powerful mean to modulate its band structure, creating synergistic active centers that promote light absorption, improve charge separation, and improve its photocatalytic efficiency for CECs removal. Co-introduction of Ni and Zr into g-C_3_N_4_, accompanied by abundant nitrogen vacancies, established multiple channels for charge migration and promoted the generation of reactive species, enabling nearly complete removal of TC within 30 min under visible light irradiation at a rate of 0.21 min^−1^, almost 10 times higher than that of pristine g-C_3_N_4_ while maintaining excellent stability in real water systems.^259^ Introducing well-dispersed Cu and Mo into g-C_3_N_4_*via* a dip-calcination route created abundant redox-active sites and improved visible-light absorption, enabling the optimized Cu/Mo@CN photocatalyst to activate persulfate and removed 97% of BPA within 60 minutes.^260^ Decorating g-C_3_N_4_ with Bi and Fe produced a catalyst that efficiently activated PMS, achieving almost complete degradation of SMX within an hour, while maintaining excellent stability.^261^

Non-metal–non-metal co-doping, although less studied, has also yielded impressive results. A tubular photocatalyst synthesized by co-doping F and P into g-C_3_N_4_ displayed accelerated electron transport and a narrowed band gap, enabling TC degradation rates up to 12 times higher than pristine g-C_3_N_4_ while maintaining 98% mineralization efficiency over repeated cycles.^262^ P and S were simultaneously incorporated into g-C_3_N_4_ through *in situ* thermal polymerization, yielding a modified photocatalyst with improved light utilization, increased surface area, and reduced electron–hole recombination.^263^ This co-doped catalyst exhibited 6 times higher degradation rate for a TC and 7 times higher degradation rate for MO, as compared to pristine g-C_3_N_4_, together with high levels of mineralization. Ganganboina *et al.* fabricated one-dimensional g-C_3_N_4_ co-doped with B and P, where the formation of P–N and B–C bonds lowered electrical resistance and suppressed charge recombination, enabling rapid visible-light degradation of DCF with excellent reusability across successive cycles.^264^ He *et al.* developed S and O co-doped g-C_3_N_4_ nanosheets containing vacancies and defects, which provided abundant accessible sites and optimized the electronic structure with band gap value of 2.64 eV for CN–S, 2.65 eV for CN–SO-v, and 2.73 eV for CN–O. The downward shift in both conduction and valence bands confirm the improved oxidative capability of CN–SO-v due to S and O co-doping, hence, this photocatalyst achieved 99% TC removal within 15 min under visible light, with a reaction rate over 9 times greater than that of the pristine g-C_3_N_4_ owing to efficient exciton generation and charge separation as shown in Fig. 10a–j.^265^

**Fig. 10:**
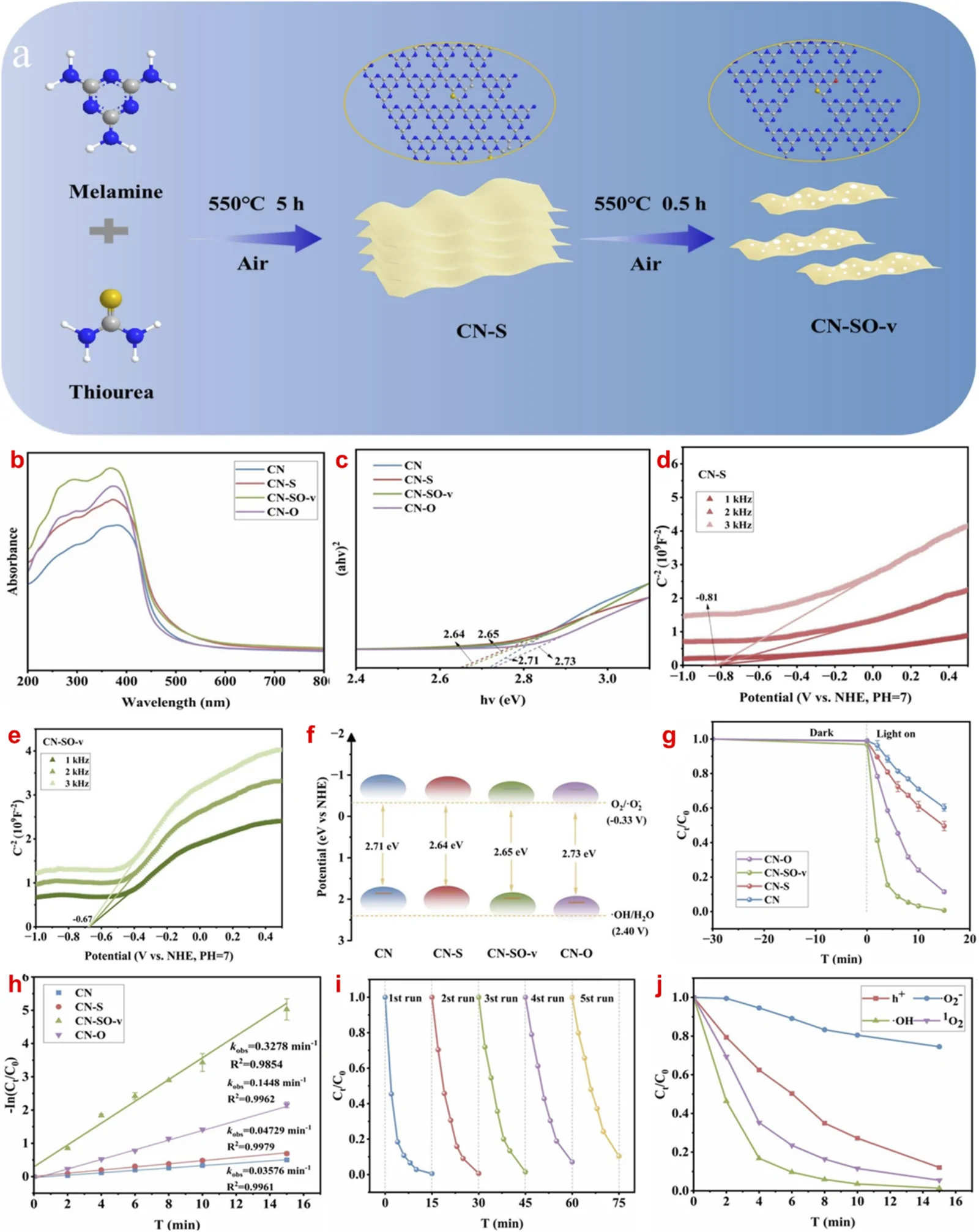
(a) Schematic illustration of the synthesis of S & O co-doped g-C_3_N_4_ (CN–SO-v) sample, (b) UV-vis DRS, (c) Kubelka–Munk plots, Mott–Schottky plots of (d) CN–S (e) CN–SO-v; (f) band structure, (g) photocatalytic degradation of TC (h) kinetics of as-prepared samples; (i) stability test of photocatalytic degradation of TC for CN–SO-v and (j) degradation efficiency of TC after adding EDTA-2Na, BQ, TBA, and TEMP as scavengers, reproduced with permission from ref. 265 Copyright 2025 Elsevier B.V.

Among the different co-doping configurations, metal–metal co-doping showed promising results, particularly in systems incorporating transition metals such as Ni, Zr, and Cu, where synergistic redox interactions facilitate rapid electron transfer and enhance reactive oxygen species formation. However, these systems may face challenges related to metal leaching and long-term stability. On the other hand, non-metal co-doping offers improved structural stability and lower environmental risk, although the absence of strong redox-active centers may limit degradation rates.

Table 5 summarizes the photocatalytic performance of g-C_3_N_4_-based materials prepared using different modification strategies. To allow a more quantitative comparison, important reaction parameters such as catalyst dose, pollutant concentration, degradation efficiency, apparent rate constant, BET surface area, and surface-area-normalized rate constant are also included.

**Table 5:** Comparative photocatalytic performance of representative modified g-C_3_N_4_-based photocatalysts toward CECs and model organic pollutants under different reaction conditions (where, vol. = volume, NR = not reported)

Photocatalyst modification	Target pollutant	Light source	Catalyst dose (g L^−1^)	Pollutant conc. (mg L^−1^)	Vol. (mL)	pH	Degradation efficiency (%)	Time (min)	Rate constant (min^−1^)	Surface area (m^2^ g^−1^)	Surface-area-normalized rate constant (g m^−2^ min^−1^)	Dominant reactive species	Ref.
**Single element doping strategy**													
B-doped g-C_3_N_4_	SMX	Visible	1.0	10	50	7	>99	30	0.201	98	0.00205	O_2_˙^−^, ˙OH, h^+^	245
S-doped 2-D g-C_3_N_4_	Phenol	Simulated solar light	0.5	10	100	NR	93	90	NR	226.9	NR	O_2_˙^−^	266
S-doped 2-D g-C_3_N_4_	TC	Visible	0.2	20	50	NR	80.9	∼120–180	NR	NR	NR	O_2_˙^−^, ^1^O_2_	267
S-doped g-C_3_N_4_	TC	Visible	0.8	25	25	∼7	98.09	40	0.01207	61.185	0.00019	O_2_˙^−^, h^+^	268
S-doped g-C_3_N_4_	TC	Solar	1.0	10	NR	4–7	98.7	60	NR	NR	NR	O_2_˙^−^, h^+^	269
S-doped g-C_3_N_4_	RhB	Visible	0.25	4.79	80	NR	97	15	0.2283	72	0.00317	O_2_˙^−^, h^+^	270
P-doped g-C_3_N_4_	SMX	Visible	1.0	10	100	NR	∼70	180	NR	NR	NR	O_2_˙^−^, h^+^	246
F-doped g-C_3_N_4_	TC	Visible	0.5	10	100	NR	99.8	180	0.0207	79.66	0.00025	O_2_˙^−^	249
F-doped g-C_3_N_4_	MB	Solar	1.0	10	50	NR	95	180	0.0120	NR	NR	˙OH, h^+^	271
C-doped g-C_3_N_4_	BPA	Visible	0.5	10	100	3–11	96	150	0.0180	82.814	0.00021	O_2_˙^−^, h^+^	250
Co-doped g-C_3_N_4_	TC	Visible	1.0	50	100	NR	97	30	0.28	28.62	0.00978	O_2_˙^−^, h^+^, ˙OH	239
Ba-doped g-C_3_N_4_	TC	Visible	1.0	20	50	10	91.94	120	0.00875	11.41	0.00076	˙OH, h^+^	272
O-doped g-C_3_N_4_	BPA	Visible	0.2	10.3	100	3–11	98	120	0.0611	70.32	0.00086	O_2_˙^−^, h^+^	273
O-doped g-C_3_N_4_	CBZ	Visible	0.1	5	100	3–11	98.7	180	0.0277	63.34	0.00043	^1^O_2_, O_2_˙^−^	274
O-doped g-C_3_N_4_	*p*-Nitrophenol	Visible	1.0	10	50	NR	99.7	120	0.0268	99.5	0.00027	O_2_˙^−^	275
	ATN			10	50		99.1	30	0.145		0.00145		
O-doped g-C_3_N_4_	Ofloxacin	Visible	1.0	10	NR	3–11	100	15	0.325	∼224	∼0.00145	O_2_˙^−^	276
	TC			10	NR		100	15	0.347		∼0.00154		
	Sulfadiazine			10	NR		100	35	0.158		∼0.00070		

**Dual element co-doping strategy**													
Ni, Zr co-doped g-C_3_N_4_	TC	Visible	1.0	10	100	NR	∼100	30	0.2097	NR	NR	O_2_˙^−^, ^1^O_2_	259
P, Mo co-doped g-C_3_N_4_	TC	Visible	0.5	10	100	NR	69	120	0.01	NR	NR	O_2_˙^−^, h^+^	254
Co, O co-doped g-C_3_N_4_	BPA	Visible	0.4	15	50	NR	100	180	0.02855	NR	NR	O_2_˙^−^	277
F, P co-doped g-C_3_N_4_	TC	Visible	0.5	10	100	3–11	98	30	0.129	13.4–16.4	∼0.00786	O_2_˙^−^, h^+^	262
Se, F co-doped g-C_3_N_4_	MB	Visible	1.0	10	50	NR	99.96	210	NR	65.451	NR	˙OH, O_2_˙^−^, h^+^	278
	TC			10	50		86.38				NR		
Na, B co-doped g-C_3_N_4_	2,4-Dichlorophenol	Visible	2.0	100	100	3.0	90.6	240	NR	NR	NR	O_2_˙^−^, h^+^	279
B, P co-doped 1-D g-C_3_N_4_	DCF	Visible	1.0	10	15	∼7	96	90	0.056	15.7	0.00356	O_2_˙^−^, h^+^	264
S, B co-doped g-C_3_N_4_	Methyl violet	Solar	1.0	20	100	∼6.4	∼85	180	0.0095	∼162	∼0.00005	˙OH, O_2_˙^−^, h^+^	280
Cu, Cl co-doped g-C_3_N_4_	TC-HCl	Visible	0.5	10	100	∼7	83.5	60	0.0271	88.4	0.0003	O_2_˙^−^, h^+^	281

**Heterojunction engineering strategy**													
g-C_3_N_4_/Fe-MOFs-10 Type-II heterojunction	Doxycycline	Visible	0.03	50	500	3–7	87.1	60	0.05523	NR	NR	˙OH, O_2_˙^−^, h^+^	282
B-doped g-C_3_N_4_/TiO_2_ Type-II heterojunction	MB	Visible	1.0	NR	NR	NR	90–95	120–240	0.06492	NR	NR	˙OH, O_2_˙^−^	283
Bi_2_WO_6_/g-C_3_N_4_ Z-scheme heterojunction	TC	Solar light	0.4	20	50	6.49	98.0	60	0.0446	80.28	0.00055	SO_4_˙^−^, O_2_˙^−^, h^+^, ^1^O_2_	284
CoS/BCN Z-scheme heterojunction	Malachite green	Visible	0.1	20	150	∼7	96.9	60	0.0542	NR	NR	O_2_˙^−^, h^+^	285
Ag_2_CrO_4_/SCN Z-scheme heterojunction	MO	Visible	0.5	15	50	∼7	90.27	20	0.10299	68.625	0.0015	O_2_˙^−^, h^+^	286
ZnO/g-C_3_N_4_ S-scheme heterojunction	Paracetamol	Visible	0.2	30	100	4–11	95	60	0.05242	11.335	0.00462	˙OH	287

**g-C**_**3**_ **N**_**4**_ **/carbon-based hybrids**													
rGO/g-C_3_N_4_ hybrid	BPA	Visible	1.0	5	40	7	∼80	240	0.056	55.8	0.001	O_2_˙^−^, ^1^O_2_	288
rGO/g-C_3_N_4_/BiVO_4_	TC-HCl	Visible	1.0	35	40	NR	72.5	150	0.00841	1.08	0.00778	O_2_˙^−^, ˙OH	289
CQDs/g-C_3_N_4_/ZnO	CIP	Sunlight simulator	1.0	10	50	NR	91.0	120	NR	NR	NR	O_2_˙^−^	290
Biochar-doped g-C_3_N_4_/CoPMoV	SMX	Visible	0.2	20	100	3–11	98.5	20	0.215	NR	NR	˙OH, h^+^, ^1^O_2_	291

**Porosity control engineering**													
Mesoporous C-doped g-C_3_N_4_	MO	Visible	0.25	10	40	NR	99.2	60	0.036	185.7	0.00019	˙OH, h^+^	292
	RhB			10	40		97.1		NR		NR		
Porous S-doped g-C_3_N_4_ rods	RhB	Visible	1.0	10	100	NR	100	120	NR	20–52	NR	˙OH, O_2_˙^−^, h^+^	293
Porous O-doped g-C_3_N_4_	RhB	Visible	0.5	10	100	NR	∼95	360	0.00643	63.2	0.0001	O_2_˙^−^	294
	TC-HCl			5	100		∼70		0.00280		0.00004		
Soft templated 3-D g-C_3_N_4_ aggregates	TC	Visible	0.5	30	100	NR	97.9	12	NR	100	NR	˙OH, O_2_˙^−^	295
Hard templated P-doped g-C_3_N_4_	TC	Visible	0.25	10	100	7	100	20	0.23367	51.98	0.0045	O_2_˙^−^, h^+^	296
3-D porous agar/CDs/g-C_3_N_4_ aerogel	Amoxicillin	Visible	0.5	3	100	NR	100	45	NR	NR	NR	O_2_˙^−^, ˙OH	297
Thin-layer B-g-C_3_N_4_ nanosheets	RhB	Visible	0.2	10	10	NR	∼100	10	0.3287	37.44	0.00877	O_2_˙^−^, h^+^	298
	Eosin Y			10	10		∼100	5	1.0885		0.02907		
	MO			10	10		94	60	0.0436		0.00116		

### 6.3 Metal oxide and semiconductor coupling

The coupling of g-C_3_N_4_ with metal oxides and other semiconductors to form a heterojunction, is an effective strategy to overcome its inherent limitations and improve its photocatalytic performance for environmental remediation.^120,299,300^ Depending on the charge transfer direction, heterojunctions of g-C_3_N_4_ mainly fall into three categories: Type-II, Z-scheme, and S-scheme systems (Fig. 11a–c).

**Fig. 11:**
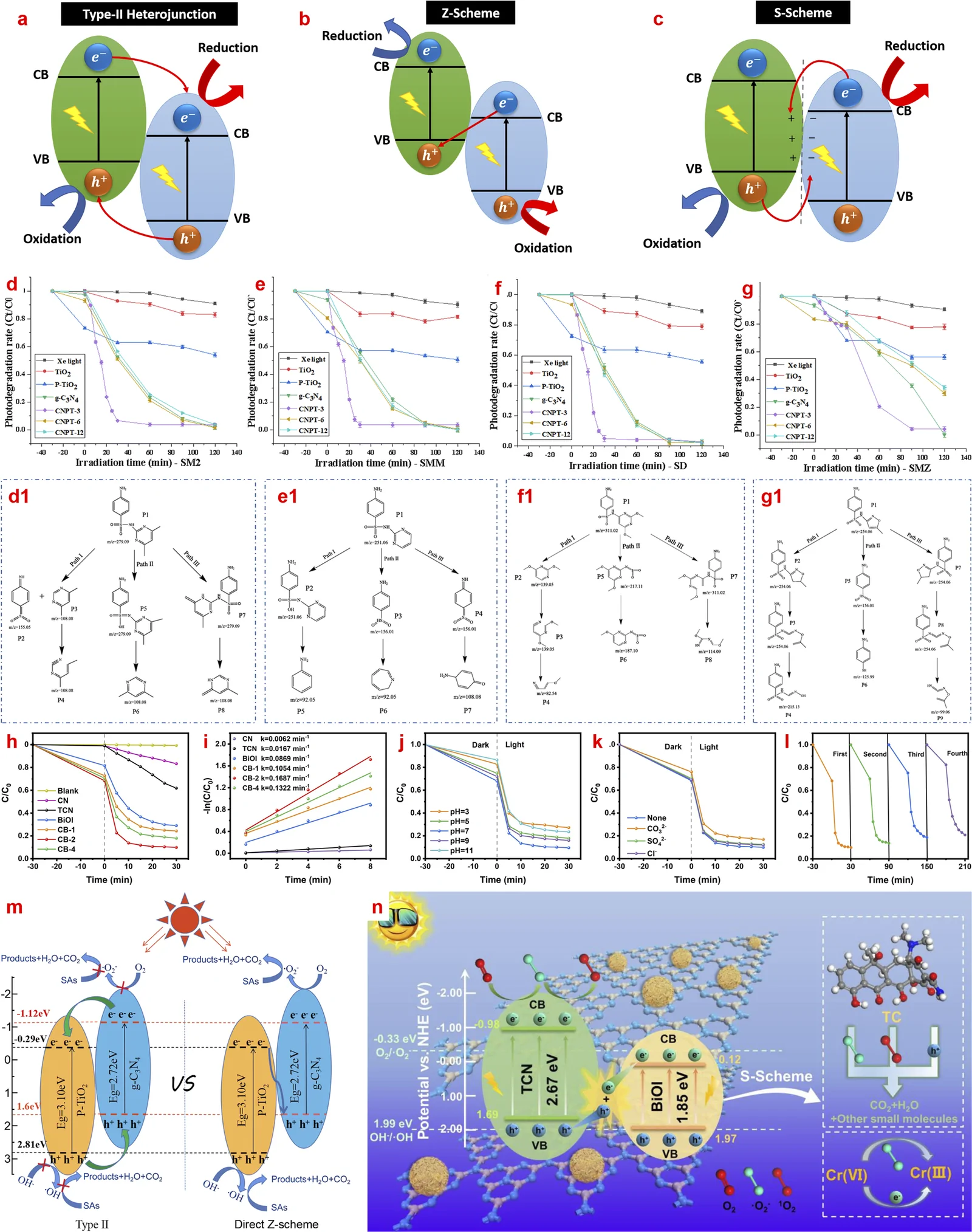
Schematic illustration of charge transfer pathways in (a) Type-II, (b) Z-scheme, and (c) S-scheme heterojunctions; photocatalytic degradation performance for four kinds of sulfonamide antibiotics at P–TiO_2_/g-C_3_N_4_ heterostructure (d) SM2, (e) SMM, (f) SD, and (g) SMZ and corresponding degradation mechanism pathways for (d1) SM2, (e1) SD, (f1) SMM, (g1) SMZ, reproduced with permission from ref. 314 Copyright 2023 Royal Society of Chemistry; (h) photodegradation activity of TC over g-C_3_N_4_/BiOI (CB-*X*, X: 1,2,4) S-scheme heterojunctions, (i) the corresponding pseudo-first-order kinetic fitted data, (j) effect of initial pH and (k) different inorganic sodium ion salts on performance of g-C_3_N_4_/BiOI photocatalysts, (l) reusability experiment of CB-2 for removal of TC, reproduced with permission from ref. 315 Copyright 2023 Elsevier B.V; comparison between (m) Type-II and direct Z-scheme photocatalytic degradation mechanism of sulfonamide antibiotics over P–TiO_2_/g-C_3_N_4_, reproduced with permission from ref. 314 Copyright 2023 Royal Society of Chemistry, and (n) S-scheme mechanism of TC over g-C_3_N_4_/BiOI, reproduced with permission from ref. 315 Copyright 2023 Elsevier B.V.

Type-II heterojunctions facilitate stepwise electron transfer, from the semiconductor with a more negative CB potential to the one with a less negative CB potential, while h^+^ migrate in the opposite direction, as shown in Fig. 11a.^301^ This configuration suppresses electron–hole recombination and improves charge carrier separation, leading to improved photocatalytic activity.^302^

Metal oxides, such as TiO_2_, ZnO, Bi_2_O_3_, BiVO_4_, Fe_2_O_3_, and SnO_2_ are commonly coupled with g-C_3_N_4_ due to their well-defined band positions, chemical compatibility, and diverse redox properties. For instance, LaNi_0.8_Fe_0.2_O_3_/g-C_3_N_4_ Type-II heterojunctions, prepared *via* a simple wet-chemical route, achieved efficient visible-light degradation of TC and reduction of Cr(vi), where Fe-induced oxygen vacancies and interfacial band alignment synergistically enhanced charge separation and stability of the catalyst.^303^ A g-C_3_N_4_/Fe-MOF Type-II heterojunction, fabricated from waste PET *via* DBD plasma, delivered a 4.4-fold boost in doxycycline removal through a photo-Fenton route, where the broadened light absorption and interfacial electron transfer between g-C_3_N_4_ and Fe-MOFs effectively suppressed charge recombination.^282^ Tian *et al.* reported the successful synthesis of BiOBr-g-C_3_N_4_ Type-II heterojunction, which degraded 87% RhB within 30 min.^304^ However, the loss of redox potential in Type-II systems often limits their efficiency in degrading complex CECs, such as pharmaceuticals and endocrine disruptors.^305^

Z-scheme heterojunctions are basically designed on the principle of photosynthesis.^306^ In a typical Z-scheme system, two semiconductors with suitable staggered band configuration are coupled together.^307–309^ In this system, the photogenerated electrons in the CB of the one photocatalyst combine with the h^+^ in the VB of the second photocatalyst, as shown in Fig. 11b. As a result, the system retains only the highly reductive electrons and highly oxidative h^+^, preserving strong redox power required for effective CECs degradation.^310^

For example, a 3-D chrysanthemum like g-C_3_N_4_/TiO_2_ Z-scheme photocatalyst, synthesized *in situ* by hydrothermal growth, achieved nearly complete TC removal within 60 min under visible light, attributed to optimized light harvesting, accelerated charge transfer, and the concerted actions of O_2_˙^−^, h^+^, and ˙OH radicals.^311^ Guo *et al.* developed an Ag@ZIF-8/g-C_3_N_4_ direct Z-scheme photocatalyst through *in situ* coprecipitation, in which Ag^+^/Ag^0^ species narrowed the ZIF-8 bandgap and together with g-C_3_N_4_, enabled 90% degradation of mixed antibiotics within 60 min under visible light through integrated adsorption–photocatalysis and efficient O_2_˙^−^/˙OH-driven redox pathways.^312^ Li *et al.* fabricated Bi_2_WO_6_/g-C_3_N_4_ Z-scheme heterojunction *via* hydrothermal synthesis, which when coupled with persulfate under solar light, achieved 98% TC removal in 60 min, with the improved electron transfer.^284^ A nanohybrid of α-Fe_2_O_3_ and g-C_3_N_4_ obtained *via* ultrasound-assisted impregnation followed by microwave conversion, formed a direct Z-scheme heterojunction, which achieved more than doubled dye degradation rates, relative to the single components, owing to the intimate interfacial bonding, efficient charge transfer, and remarkable stability over repeated cycles.^313^ Dai *et al.* engineered a direct Z-scheme P–TiO_2_/g-C_3_N_4_ heterostructure *via* calcination, achieving approximately 99% degradation (Fig. 11d–g) of four sulfonamide antibiotics including sulfamethazine (SM2), sulfadiazine (SD), sulfamonomethoxine (SMM) and SMZ within 90 min under simulated sunlight, owing to efficient interfacial charge transfer and preserved redox potentials.^314^ The Fig. 11d1–g1 display the possible photocatalytic degradation pathways for SM2, SD, SMM and SMZ, respectively. The P–TiO_2_/g-C_3_N_4_ heterojunction follow a direct Z-scheme mechanism as compared to P–TiO_2_ which follows Type-II mechanism, therefore, the P–TiO_2_/g-C_3_N_4_ heterostructure showed enhanced charge separation and reduced electron–hole recombination, compared to pristine P–TiO_2_. In Type-II comparison, the conventional charge transfer pathway would weaken the redox capability, whereas the direct Z-scheme retains the strong reduction potential of g-C_3_N_4_ electrons and oxidation potential of P–TiO_2_ holes. Electrons in the CB of g-C_3_N_4_ reduce O_2_ to O_2_˙^−^, while holes in the VB of P–TiO_2_ generate ˙OH radicals from hydroxyl ion (OH^−^), both contributing to efficient pollutant photocatalytic degradation (Fig. 11m).

S-scheme heterojunction is formed by coupling a reduction photocatalyst (RP) and an oxidation photocatalyst (OP), where the RP possesses relatively higher Fermi level, conduction CB, and VB positions than the OP, as shown in Fig. 11c.^316,317^ In S-scheme systems, charge transfer is driven by band bending and an internal built-in electric field at the interface.^318^ Selective recombination occurs only between carriers possessing weak redox potential, while high-energy electrons and h^+^ remain separated on opposite sides of the interface.^319^ Thus, S-schemes combine efficient charge separation with maximized oxidation and reduction capability, similar to Z-schemes but without requiring electron mediators or externally coupled structures.

A dual S-scheme g-C_3_N_4_/Ag_3_PO_4_/g-C_3_N_4_ photocatalyst, synthesized *via* controlled decomposition, achieved a 4.4-fold increase in TC removal by activating molecular oxygen at two reactive sites, where the dual S-scheme pathway and internal field suppressed charge recombination and enhanced ROS generation.^320^ Alsalme *et al.* designed a S-scheme Ag_2_CrO_4_/g-C_3_N_4_ heterojunction through sonochemical assembly, where uniformly anchored Ag_2_CrO_4_ nanoparticles enhanced solar absorption and enabled 96% RhB degradation under solar irradiation *via* h^+^ and O_2_˙^−^ oxidation pathways.^321^ An S-scheme ZnO/g-C_3_N_4_ composite, optimized at 10 wt% g-C_3_N_4_ achieved 95% paracetamol removal within 60 min under visible light, with optimized band gap alignment and rapid interfacial charge migration, resulting in exceptional activity and durability.^287^ Gu *et al.* constructed an S-scheme g-C_3_N_4_/Bi_8_(CRO_4_)O_11_ heterojunction by self-assembly, in which optimized 10 wt% g-C_3_N_4_ delivered markedly higher degradation rates of norfloxacin and BPA under visible light, up to 2.3 and 9.1 times greater than that of pristine components through efficient S-scheme charge transfer and suppressed carrier recombination.^322^ Dou *et al.* engineered tubular g-C_3_N_4_/BiOI S-scheme heterojunctions, where the optimized CB-2 composite achieved 90.1% TC and 96.8% Cr(vi) removal (Fig. 11h–l & n), with DFT-supported S-scheme charge transfer.^315^ The formation of a tight interface between tubular g-C_3_N_4_ and BiOI is essential to generate an internal electric field that drives S-scheme charge migration.

### 6.4 g-C_3_N_4_/carbon-based hybrids

Hybridization of g-C_3_N_4_ with carbon-based nanomaterials such as graphene, reduced graphene oxide (rGO), carbon nanotubes (CNTs), carbon dots (CDs), and porous carbon frameworks, has emerged as one of the most promising techniques to improve its photocatalytic activity for CECs degradation.^31,323–326^ These carbon materials introduce highly conductive networks, improve charge carrier mobility, enhance visible-light absorption, and offer abundant active sites for CECs adsorption.^327–329^

Among these, rGO is most extensively studied. When coupled with g-C_3_N_4_, *via* thermal annealing, electrostatic self-assembly, or hydrothermal synthesis, rGO functions as a conductive platform and electron sink.^330,331^ Moreover, the hydrophilic oxygen-containing groups on rGO facilitate strong π–π stacking and hydrogen bonding with aromatic CECs, increasing adsorption and reaction probability.^332–334^ An all-solid-state rGO/g-C_3_N_4_/BiVO_4_ Z-scheme heterostructure, synthesized hydrothermally, achieved markedly higher TC degradation rate than its binary counterparts, as the Z-scheme and rGO mediator synergistically accelerated charge separation and boosted photocatalytic efficiency.^289^ Zhang *et al.* developed a 2-D/2-D-rGO/Ag/g-C_3_N_4_ heterostructure, in which Ag plasmonic effects and rGO's electron-sink capability synergistically promoted charge separation, enabling >97% TC removal within 60 min under LED light and extending activity to other antibiotics.^335^ Zhao *et al.* fabricated a 3-D porous g-C_3_N_4_/rGO composite in which the interconnected architecture and conductive rGO sheets enhanced charge migration and adsorption capacity, achieving 96% RhB removal through efficient electron–hole separation.^336^ Embedding rGO into g-C_3_N_4_ produced a metal-free photocatalyst that tripled the photodegradation rate of BPA, achieving 80% mineralization in 4 h through enhanced charge separation and O_2_˙^−^/^1^O_2_ mediated oxidation with excellent stability in complex waters (Fig. 12a–d).^288^

**Fig. 12:**
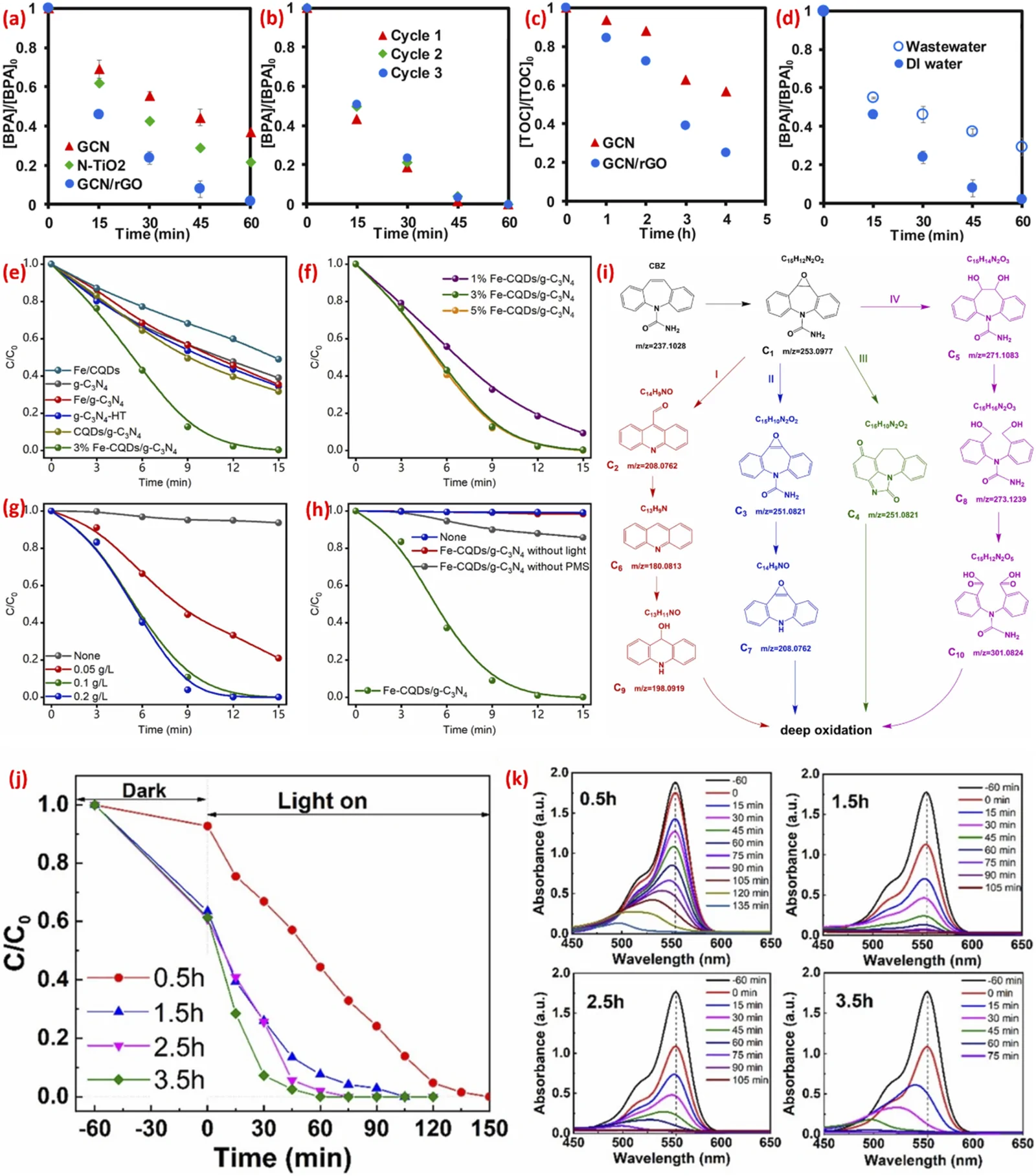
Photocatalytic degradation of BPA (a) performance comparison between GCN, N-doped TiO_2_ and GCN/rGO-0.25% under visible light, (b) cyclic stability, (c) TOC and (d) the BPA, reproduced with permission from ref. 288 Copyright 2021 Elsevier B.V. Photocatalytic degradation of carbamazepine under solar light in the presence of PMS (e) comparison of different catalysts, (f) effect of Fe loading in Fe-CQDs/g-C_3_N_4_, (g) catalyst dosage effect, (h) control experiments confirming the requirement of both light and PMS and (i) possible degradation mechanism and intermediates of CBZ degradation, reproduced with permission from ref. 337 Copyright 2024 Elsevier B.V. (j) Photocatalytic degradation of RhB under solar irradiation using g-C_3_N_4_ nanosheets synthesized with different annealing durations, (k) UV-visible absorption spectra of RhB at corresponding irradiation times, reproduced with permission from ref. 338 Copyright 2021 Elsevier B.V.

Similarly, carbon nanotubes offer 1-D conductive pathways that bridge photoactive domains. Multi-walled CNTs (MWCNTs) and single-walled CNTs (SWCNTs) integrated with g-C_3_N_4_ demonstrated faster interfacial charge transfer, minimized recombination, and increased mechanical strength for photoreactors.^339,340^ These composites outperform pristine g-C_3_N_4_ in degrading mixed pharmaceuticals and exhibit superior photostability during multi-cycle use.^31^ For example, WO_3_@g-C_3_N_4_@MWCNT composite degraded approximately 79.5% TC in 120 min under visible light, with MWCNTs acting as an electron bridge between g-C_3_N_4_ and the WO_3_ to suppress recombination as confirmed by radical trapping and electron microscopy studies.^341^ In another study, g-C_3_N_4_/CNT/BiVO_4_, achieved approximately 80.6% phenol removal in 120 min under solar light with optimized CNT loading and use of H_2_O_2_, showing that CNTs increase rate and improve interfacial contact.^342^

Carbon quantum dots (CQDs) provide both up-conversion photoluminescence and electron-accepting functionality.^343–346^ They absorb in the UV-NIR range and can transfer electrons to the CB of g-C_3_N_4_, extending activity into the near-infrared spectrum.^347,348^ For instance, CQD-modified layered g-C_3_N_4_/flower-like ZnO heterojunctions, optimized at 3 wt% CQDs, achieved 91% CIP removal within 120 min under simulated sunlight, as the CQDs enhanced charge transfer and O_2_˙^−^ mediated oxidation while maintaining excellent stability over repeated cycles.^290^ Tubular g-C_3_N_4_ modified with CQDs delivered a 5-fold enhancement in CBZ degradation under visible light, as CQDs suppressed charge recombination and promoted O_2_˙^−^ and h^+^ oxidation, with DFT-supported pathway analysis confirming selective radical attack and excellent stability.^349^ In another study, CQDs decorated on in-tubular g-C_3_N_4_ grown on carbon cloth achieved 98% CIP degradation within 60 min under visible light, as the CQD-g-C_3_N_4_ heterojunction enhanced light harvesting and carrier separation while the carbon cloth support afforded easy recovery and stability.^350^ Liu *et al.* synthesized Fe-CQDs/g-C_3_N_4_*via* a one-step hydrothermal route, where Fe–N coordination and CQD decoration narrowed the band gap and promoted charge separation, enabling complete CBZ removal within 15 min under solar light with PMS activation.^337^ The influence of Fe incorporation and CQD decoration on photocatalytic activity is reflected in the accelerated degradation kinetics shown in Fig. 12e–h. The optimal 3 wt% Fe-CQDs/g-C_3_N_4_ achieved complete CBZ removal within 15 min, while control experiments confirmed that both light irradiation and PMS were essential to drive the reaction. Different possible pathway for the degradation of CBZ are also shown in Fig. 12i.

Porous carbon and biomass-derived carbon have also been employed as supports for g-C_3_N_4_ nanosheets. Their large surface area, porosity, and high adsorption affinity allow the concentration of pollutants near the active sites and promote efficient degradation.^351,352^ For instance, Yang *et al.* designed biochar-doped g-C_3_N_4_ coupled with CoPMoV polyoxometalates, forming a CCN/CoPMoV composite that narrowed bandgap and reduced the recombination of charge carrier, enabling 98.5% SMX removal within 20 min in a PMS/visible-light system through ˙OH, h^+^, and ^1^O_2_ oxidation with excellent recyclability.^291^ An activated biochar/K^+^-doped g-C_3_N_4_ composite (ACB-K-g-C_3_N_4_), prepared by ultrasonic milling, showed 4-fold higher degradation rates for RhB, TC, norfloxacin, and chloramphenicol than K-g-C_3_N_4_, as its porous, defect-rich architecture enhanced absorption, accelerated electron transfer, and promoted O_2_˙^−^, h^+^, and ˙OH generation with strong disinfection and stability.^353^ Wang *et al.* developed a biochar/g-C_3_N_4_/BiOBr heterojunction, where the optimized 0.75-BCN. BiOBr achieved 86.5% TC removal in 72 min under visible light, as incorporation of biochar enhanced adsorption of contaminants, leading to enhanced degradation efficiency.^354^ Graphene-like *Enteromorpha* biochar-modified g-C_3_N_4_ activated PMS under visible light, achieving approximately 90% TC removal in 1 h, as biochar-induced π–π interactions and electron withdrawing groups improved charge separation and favored O_2_˙^−^/^1^O_2_ oxidation with broad environmental tolerance.^355^ A crawfish-shell biochar/g-C_3_N_4_ composite prepared by co-pyrolysis achieved optimal enrofloxacin degradation at 2% biochar loading under visible light, as biochar-CN synergy enhanced charge separation and promoted O_2_˙^−^ attack on the piperazine ring.^356^

### 6.5 Morphological engineering

Morphological engineering is another promising technique that focuses on tailoring the external architecture and dimensionality of g-C_3_N_4_ to enhance light utilization, charge transport, and active-site accessibility.^357,358^ Transforming bulk g-C_3_N_4_ into 2-D nanosheets, 3-D aerogels, hollow spheres, or hierarchical microspheres reduces charge migration distance and increases the exposure of catalytic surfaces, which is particularly advantageous for efficient removal of trace-level CECs.^338,359,360^

Thermal exfoliation is one of the frequently used strategies, in which layered g-C_3_N_4_ is delaminated into few-layer nanosheets with higher surface exposure and improved electron mobility.^361,362^ For instance, ZnWO_4_ coupled with thermally exfoliated g-C_3_N_4_ nanosheets (CNNs) showed maximum photocatalytic activity at 30 wt% CNNs, achieving efficient SMX degradation through h^+^ and O_2_˙^−^ oxidation while preserving high stability over multiple cycles.^363^ Ultrathin g-C_3_N_4_ nanosheets were obtained through post-thermal exfoliation of bulk urea-derived g-C_3_N_4,_ resulting in significantly increased surface area and an extended visible-light absorption range. These structural changes improved charge separation and light harvesting, leading to markedly enhanced photocatalytic degradation of organic pollutants as compared to bulk g-C_3_N_4_.^38^ Wang *et al.* reported the synthesis of g-C_3_N_4_ nanosheets, with increased surface area by extending the annealing duration during melamine condensation, allowing the layered stacks to delaminate into thinner sheet-like architectures.^364^ The optimized nanosheets displayed a slightly narrowed band gap and enhanced charge separation efficiency, which facilitated visible-light photocatalysis, achieving complete RhB degradation within 60 min under simulated visible-light irradiation. Similarly, Linh *et al.* demonstrated the synthesis of ultrathin g-C_3_N_4_ nanosheets *via* one-step thermal polycondensation of urea, where the annealing duration (0.5–3.5 h) controlled the morphology evolution from stacked layers to ultrathin nanosheets. The prolonged calcination promoted thermal exfoliation and increased the BET surface area from 52.1 to 101.7 m^2^ g^−1^, narrowed the band gap from 2.63 to 2.52 eV, and improved charge separation efficiency. The optimized ultrathin 3.5 h sample showed nearly complete RhB degradation within 60 min under simulated solar irradiation as shown in Fig. 12j and k.^338^

Other advanced architectures include 3-D porous networks, hollow spheres, nanorods, nanotubes, and aerogels. 3-D aerogels formed *via* freeze-drying or hydrothermal assembly combine low density with high porosity, allowing enhanced light scattering, long residence times for pollutants, and rapid charge transport. Agar/CDs/g-C_3_N_4_ aerogels with a 3-D porous network achieved complete amoxicillin removal within 45 min under light, outperforming g-C_3_N_4_ and agar-based counterparts, which can be correlated with combined action of agar adsorption, carbon-dot light harvesting, and g-C_3_N_4_ catalysis.^297^ 3-D g-C_3_N_4_ encapsulated in cellulose/graphene-oxide aerogels achieved complete MB removal within 90 min, as the hybrid's microporous network, high surface area, and improved conductivity enabled efficient adsorption with excellent recyclability.^365^ WO_3_/g-C_3_N_4_ hollow microspheres synthesized *via in situ* hydrolysis-polymerization offered a well-defined Z-scheme interface, where the cavity architecture trapped photons and maximized exposure of redox-active sites, enabling markedly improved degradation activity of TC and ceftiofur sodium.^235^ Fe-modified g-C_3_N_4_ nanorod bunches synthesized *via* chemical vapor co-deposition displayed a well-organized rod-like architecture that enhanced light harvesting and charge transport. The Fe coordination within the g-C_3_N_4_ lattice prolonged carrier lifetimes and together with the high aspect-ratio rods, enabled 82.5% oxytetracycline degradation with excellent cycling stability.^366^

### 6.6 Porosity control

Bulk g-C_3_N_4_ is commonly prepared by thermal polycondensation of melamine or dicyandiamide. It usually has a low surface area (<10 m^2^ g^−1^) and closely packed layers. This limits contact between the catalyst and pollutants and can also increase charge-carrier recombination.^130,367–369^ Porosity control can overcome some of these problems. It increases the surface area and changes the pore size by introducing micropores, mesopores, or macropores into g-C_3_N_4_.^200,370–372^ The resulting pores can improve reactant diffusion and provide more accessible catalytic sites for pollutant degradation.

Thermal exfoliation is one of the simple methods used to prepare porous g-C_3_N_4_. During thermal treatment, pores can form and more surface becomes exposed. In one study, porous g-C_3_N_4_ nanosheets prepared by thermal exfoliation of bulk g-C_3_N_4_ had a surface area of 212 m^2^ g^−1^. The material degraded more than 95% of methyl-, ethyl-, and propylparaben under solar light. Its better performance than bulk g-C_3_N_4_ was related to the larger exposed surface and efficient O_2_˙^−^ generation.^373^

Supramolecular self-assembly is another method used to prepare porous g-C_3_N_4_.^374,375^ In this method, nitrogen-rich precursors first arrange into supramolecular structures through non-covalent interactions, mainly hydrogen bonding. These pre-arranged structures are then thermally polymerized to produce porous g-C_3_N_4_. The resulting structure can have a larger accessible surface area, interconnected pores, more exposed active sites, better mass transfer, and improved light utilization.^375–377^ The interconnected pores are also useful for CEC degradation because they can help pollutants reach the available active sites. Recently, Bai *et al.* prepared 3-D spongy porous g-C_3_N_4_ by supramolecular self-assembly using melamine and oxalamide.^179^ The optimized sample, SC1, had a surface area of 59.41 m^2^ g^−1^, compared with 9.65 m^2^ g^−1^ for directly calcined g-C_3_N_4_. Its porous structure also improved visible-light utilization and charge separation. SC1 degraded 88.8% of TC within 60 min and showed a rate constant of 0.0740 min^−1^. In comparison, bulk g-C_3_N_4_ showed 73.9% degradation with a rate constant of 0.0346 min^−1^. SC1 still showed about 80% degradation after ten cycles, indicating good stability. In another study, Cu-doped porous g-C_3_N_4_ was prepared by supramolecular self-assembly.^378^ Its surface area increased 13-fold to 142.8 m^2^ g^−1^, together with a broader visible-light response. The ordered mesoporous structure and Cu sites improved charge transfer and resulted in rapid and recyclable TC photodegradation. Zhou *et al.* also used supramolecular self-assembly to prepare coral-like porous Na-doped g-C_3_N_4_ (NaCCN), using melamine and cyanuric acid.^379^ NaCCN completely degraded TC-HCl within 18 min and showed a rate constant of 0.224 min^−1^, which was 32 times higher than that of pristine g-C_3_N_4_. The material also maintained high activity for 10 cycles. The improved activity was related to better visible-light absorption, more active sites, and improved charge separation and transfer.

Metal ions can also be added during supramolecular self-assembly to control the structure of g-C_3_N_4_. Chen *et al.* used Ag(i) to control the interaction between melamine and cyanuric acid during self-assembly.^374^ The material was then calcined at 550 °C to produce Ag-containing 3-D hierarchical carbon nitride (Fig. 13a). Ag(i) introduced N–Ag–N coordination interactions into the supramolecular structure and affected the final assembly. The SEM and TEM images in Fig. 13b–f show a 3-D porous structure formed by wrinkled and arranged nanosheets. Small Ag nanoparticles were distributed throughout the carbon nitride and were mainly below 5 nm in size. A lattice spacing of about 0.24 nm was assigned to the Ag (111) plane (Fig. 13g and h). Elemental mapping also showed the distribution of C, N, and Ag throughout the porous structure (Fig. 13i–l).

**Fig. 13:**
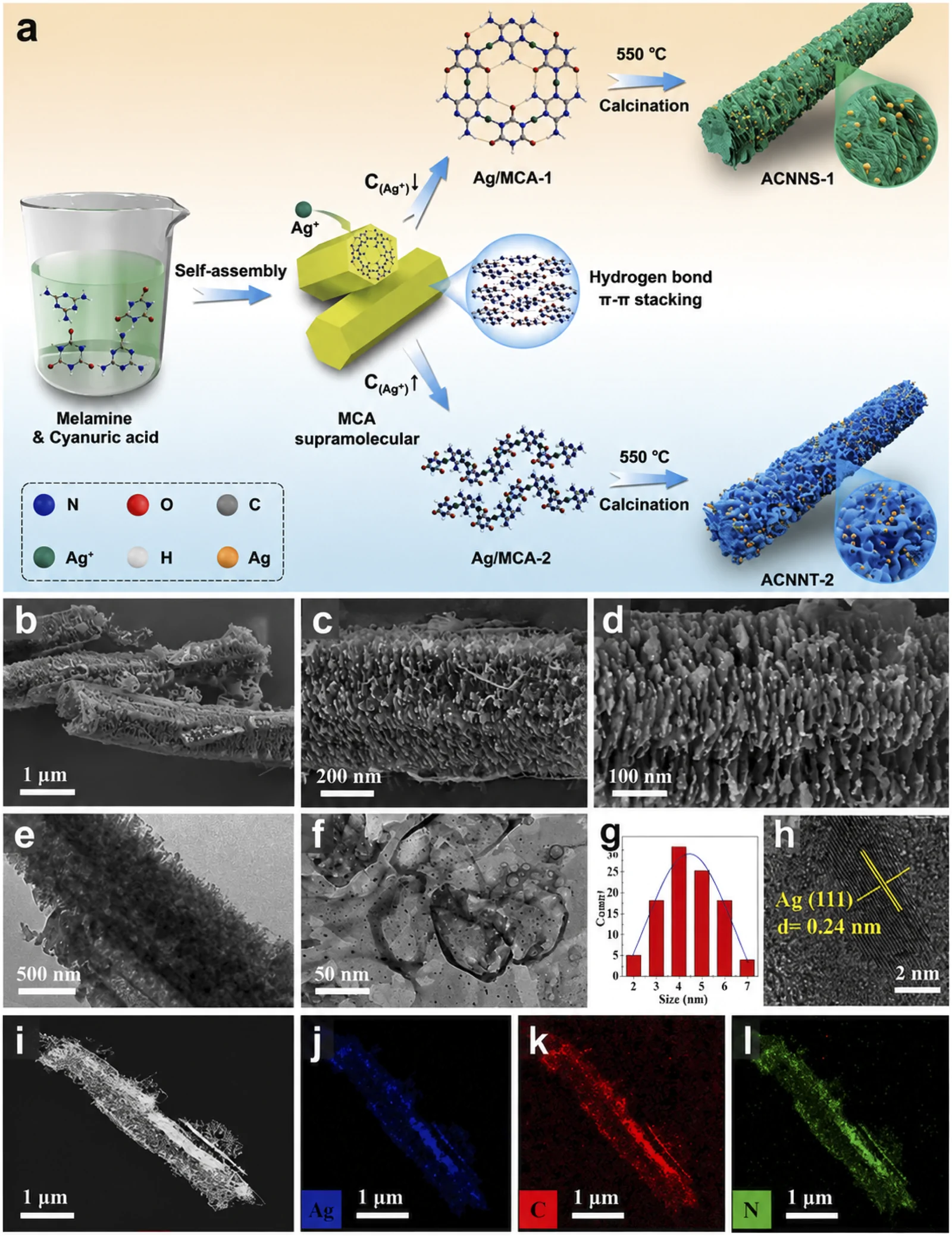
(a) Schematic representation of the preparation of hierarchical carbon nitride (ACNNS) through Ag(i)-controlled coordination with CM precursors, (b–d) SEM images and (e and f) TEM images of ACNNS-1, (g and h) particle-size distribution and HRTEM analysis of Ag nanoparticles supported on ACNNS-1, (i–l) HAADF-STEM image and corresponding elemental mapping of ACNNS-1, reproduced with permission from ref. 374 Copyright 2022, Elsevier.

Hard templating strategies for g-C_3_N_4_ often employ silica-based templates, such as colloidal SiO_2_, nanoparticles or mesoporous silica like SBA-15.^110,380^ P-doped tunnel-shaped g-C_3_N_4_ synthesized with SBA-15 as a hard template and phosphonitrilic chloride trimer as P source achieved a rate constant of 0.234 min^−1^ for TC degradation, which is 3 and 30 times higher than undoped and non-templated g-C_3_N_4_ owing to enhanced O_2_˙^−^ generation and suppressed electron–hole recombination.^296^ Tunnel-structured g-C_3_N_4_ synthesized *via* vapor deposition with SBA-15 as a hard template showed markedly enhanced activity, with SBA-15-CN delivering a 10-fold higher rate for TC degradation than bulk g-C_3_N_4_, owing to its mesoporous channels, abundant N vacancies, and efficient h^+^/O_2_˙^−^ driven photodegradation.^381^

Similarly, soft-templated methods using supramolecular aggregates, such as hexadecyl trimethyl ammonium chloride, enable the formation of porous g-C_3_N_4_ structures with interconnected mesopores that facilitate molecular diffusion and light penetration.^149^ Unlike direct supramolecular self-assembly of molecular precursors, soft-templating methods use removable organic structure-directing agents to generate interconnected porous frameworks. 3-D g-C_3_N_4_ aggregates composed of hollow bubbles, obtained by hydrothermal treatment of a supramolecular precursor with Pluronic P123 followed by annealing, delivered 97.9% TC degradation in 12 min under visible light, as the porous architecture enhanced light absorption, charge separation, and O_2_˙^−^/˙OH oxidation.^295^ A TiO_2_/SiO_2_/g-C_3_N_4_ hybrid fabricated *via* ternary soft-template route (UTSCN) achieved a 2.63-fold higher berberine removal in sea water than g-C_3_N_4_, as its flower-like porous morphology and SiO_2_ component enabled strong adsorption coupled with efficient visible-light photocatalysis.^382^

A dual-template strategy combining soft and hard templates enabled simultaneous doping and mesoporous structure formation in g-C_3_N_4_, significantly enhancing photocatalytic performance by increasing active site density and promoting efficient charge transfer. For instance, mesoporous Ag_2_O/g-C_3_N_4_ heterostructures fabricated with Pluronic 31R1 and MCM-41 templates achieved complete CIP degradation within 60 min under visible light, which is 4 and 10 times faster than Ag_2_O and g-C_3_N_4_ alone, as uniformly dispersed 4 nm Ag_2_O nanoparticles, high surface area, and strong interfacial coupling enhanced charge separation and durability.^383^ C-doped mesoporous g-C_3_N_4_ nanosheets were synthesized through a combined soft-hard templating route, enabling a substantial increase in surface area and pore volume. The optimized sample (PCN-1) exhibited enhanced visible-light absorption and more efficient charge separation, achieving ∼99% MO and ∼97% RhB degradation within 120 min while maintaining excellent cycling stability, highlighting its potential for cost-effective industrial wastewater treatment.^292^

However, both hard and soft templating require template removal steps. To address scalability, salt-templated or molten salt methods have emerged as highly scalable techniques that offer controlled crystallinity, pore size modulation, and morphology adjustment without hazardous etchants.^158,384^ 3-D foam-like g-C_3_N_4_ obtained by salt-templated thermal polycondensation exhibited a surface area 12 times higher than that of bulk material and delivered a 64-fold faster TC degradation rate, as its hierarchical pores and surface functionalities enhanced charge migration and ˙OH/^1^O_2_/O_2_˙^−^ driven oxidation.^384^ g-C_3_N_4_ prepared *via* NaCl-assisted molten-salt treatment of melamine (NaMe-*x*) achieved a 2.1-fold higher TC hydrochloride (TC-HCl) degradation rate than pristine g-C_3_N_4_, as the tailored microstructure promoted oxidation while maintaining low toxicity toward most aquatic organisms.^194^Table 6 offers a comparative analysis highlighting the advantages, practical limitations, and commonly used examples of each modification approach discussed above, providing insights into their suitability and effectiveness for real-world applications.

**Table 6:** Decision matrix for selecting g-C_3_N_4_ modification strategies for photocatalytic degradation of CECs based on physicochemical effects, mechanistic trade-offs, and target-contaminant characteristics^a^

Modification strategy	Primary physicochemical property modified	Key advantages for CEC degradation	Mechanistic trade-offs and limitations	Target CEC characteristics	Representative CECs
Doping	Band structure, defects, surface chemistry	Light absorption, charge separation, improves ROS generation	Recombination centers, band shifts, leaching	Hydrophilic or ionizable CECs	TC,^375^ SMX,^385^ CIP,^386^ BPA^387^
Co-doping	Electronic structure, defects, surface charge	Band tuning, charge separation, multiple reactive sites	Complex dopant interactions, recombination, difficult optimization	Recalcitrant hydrophilic or moderately hydrophobic CECs	TC,^259^ DCF,^388^ BPA,^260,389^ SMX^390^
Type-II heterojunction	Band alignment, charge transfer	Charge separation, reduced charge recombination	Lower redox ability, limited ROS	Hydrophilic CECs	Doxycycline,^391^ TC^392^
Z-scheme heterojunction	Charge transfer, redox potential	Charge separation, strong redox ability	Requires suitable band alignment and efficient interfacial transfer, mechanism requires rigorous experimental verification	Recalcitrant CECs requiring strong oxidation/reduction	TC,^393,394^ SMX,^395^ parabens^396^
S-scheme heterojunction	Interfacial electric field, band bending, charge separation	Charge separation, strong redox ability	Requires appropriate Fermi-level/band alignment, mechanism is often difficult to verify conclusively	Hydrophilic or ionizable CECs	Parabens,^397,398^ acetaminophen,^399^ norfloxacin,^400^ BPA^401^
Carbon-based hybrids	Electrical conductivity, adsorption, charge transport	Electron transport, adsorption, π–π interactions can favor aromatic pollutants	Light shielding, stability, charge recombination	Aromatic and relatively hydrophobic CECs	Azoxystrobin (pesticide),^402^ BPA,^403^ CIP,^404^ CBZ,^405^ SMX^406,407^
Morphological engineering	Morphology, surface area, transport distance	More active sites, shortens carrier migration distance, light utilization	Structural disorder, compromised stability, charge recombination	Bulky or diffusion-limited CECs, systems requiring greater catalyst-pollutant contact	TC,^408^ SMX,^409^ CBZ^410^
Porosity engineering	Surface area, porosity, mass transfer	Increases adsorption and active site accessibility, improves mass transfer	Pore blockage, limited mineralization	Moderately hydrophobic or size-compatible CECs	PCB 180,^411^ TC,^179^ CIP,^409^ SMX^412,413^

a The suitability of a modification strategy for a particular CEC depends on several factors, including hydrophobicity (log *K*_ow_), p*K*_a_ and ionization state, molecular size, aromaticity, functional groups, and solution pH. These factors influence the interaction of CECs with the photocatalyst surface and their subsequent degradation.

Based on these findings, the design of g-C_3_N_4_ photocatalysts should be guided by the properties of the target CEC rather than by maximizing a single material parameter. Broader light absorption should be balanced with preservation of sufficient redox potential for ROS generation. Surface area and porosity should be selected according to pollutant size, hydrophobicity, and adsorption behavior, while dopants should be chosen according to the desired changes in electronic structure, surface interaction, and degradation pathway. Excessive doping or defect formation should be avoided because it may introduce recombination centers.

## 7 Transformation pathways, intermediate toxicity, and mineralization of CECs over g-C_3_N_4_-based photocatalysts

One of the important aspect about CEC photodegradation is that the complete removal of parent pollutant molecule does not mean the complete mineralization or suitability of the photocatalytic treatment. During degradation, the pollutant molecules transform into several intermediates and degradation products, which are sometimes even more toxic than the parent molecule. Therefore, it is necessary to identify the toxicity of transformation intermediates as well as the final products, along with the mineralization rates to evaluate the feasibility of the photocatalytic system. This section discusses these aspects for representative CECs degraded using g-C_3_N_4_-based photocatalysts. Furthermore, a comparative summary of various transformation pathways, toxicity changes, and extent of mineralization for representative CECs. Is provided in Table 7.

**Table 7:** Reported transformation products, degradation pathways, toxicity evolution, and mineralization of representative CECs using g-C_3_N_4_-based photocatalysts

CEC	Photocatalyst	Dominant ROS	Major molecular transformation	Key intermediates	Toxicity assessment	Toxicity evolution	Percent degradation	TOC removal (%)	Ref.
TC	Cyano-deficient g-C_3_N_4_/Sr_2_MgSi_2_O_7_:Eu^2+^, Dy^3+^ (6% CCN@SMSED)	O_2_˙^−^, h^+^, ^1^O_2_, ˙OH minor under light	Demethylation, deamination, and dihydroxylation	Fifteen transformation products (P1–P15)	TEST/(QSAR): acute toxicity, BCF, developmental toxicity and mutagenicity; *S. aureus* growth assay	Decreased	70.47% after 1 h light; 85.87% after subsequent 12 h dark	NR	414
TC-HCl	NaCl-modified g-C_3_N_4_ (NaMe-1)	˙OH	Dehydration, demethylation, functional-group loss, hydroxyl attack/oxidation and ring-opening reactions	Seven transformation products (P1–P7)	ECOSAR prediction (fish, *Daphnia* and green algae) + *Chlorella vulgaris* growth/chlorophyll assay + *Vibrio fischeri* luminescence assay	Mixed	94.36% after 120 min	NR	422
TC-HCl	CaCl_2_-modified g-C_3_N_4_ (CaCN-1)	O_2_˙^−^	Dehydration followed by demethylation and dihydroxylation	Seven transformation products (P1–P7)	Experimental acute-toxicity assays using *Chlorella vulgaris* and *Vibrio fischeri*	Decreased	97.65%	66.96% TOC removal	423
CBZ	ZnFe_2_O_4_/(P,S)-g-C_3_N_4_/2-D TiO_2_ (10%) + PS	O_2_˙^−^	Electron-transfer oxidation, hydroxylation, deamidation, ring contraction and further oxidation	Six transformation products (P1–P6)	NR	NR	>99.5% after 15 min	74% TOC removal after 120 min	416
BPA	CaCN_2_-templated modified g-C_3_N_4_ (MCN-0.25)	O_2_˙^−^	Hydroxylation, C–C bond cleavage, fragmentation, and further oxidation to smaller organic acids	Eight transformation products (P1–P8)	ECOSAR-predicted acute and chronic toxicity for fish, *Daphnia*, and green algae	Mostly decreased	100% after 40 min	NR	418
BPA	g-C_3_N_4_/Bi_4_O_5_Br_2_ Z-scheme heterojunction	O_2_˙^−^, h^+^	Hydroxylation, bond cleavage, and ring opening	Seven transformation products (P1–P7)	NR	NR	98% after 80 min	78.8%	417

### 7.1 Tetracycline (TC)

TC is a widely studied antibiotic in g-C_3_N_4_ photocatalytic systems due to its multiple amine, hydroxyl, and carbonyl functionalities that can be attacked during photocatalytic degradation. For example, Hu *et al.* demonstrated the successful degradation of TC over a round-the-clock photocatalyst cyano-deficient g-C_3_N_4_/Sr_2_MgSi_2_O_7_:Eu^2+^, Dy^3+^ (CCN@SMSED) and reported the formation of 15 transformation products, which were identified using LC-MS.^414^ Demethylation, deamination, and dihydroxylation were proposed as major transformation pathways. In the first pathway, TC (*m*/*z* 445) underwent demethylation to form a product at *m*/*z* 416, which was further degraded through deamination, dehydration, and ring opening to form smaller products. On the other hand, second pathway showed the formation of a product at *m*/*z* 342, which was further converted into products at *m*/*z* 282 and 233. A third pathway involved dihydroxylation, producing a product at *m*/*z* 460, and its breakdown into smaller molecules. Further oxidation eventually produced several low-molecular-weight intermediates. Direct electron transfer was also involved in TC degradation. These results show that TC can degrade through different pathways during photocatalytic degradation. Toxicity was assessed through TEST/QSAR predictions, as well as *Staphylococcus aureus* growth assay. The results showed that most degradation products were predicted to be less toxic than TC, although some products still showed higher toxicity. The *Staphylococcus aureus* test also showed that bacterial growth improved during treatment, indicating that the overall toxicity decreased. Mineralization of TC has also been evaluated using TOC analysis. Pattanayak *et al.* reported about 55% degradation of tetracycline hydrochloride by g-C_3_N_4_ after 60 min, while TOC removal reached about 52%.^415^ Since TOC measures the remaining organic carbon in the solution, the lower TOC value shows that degradation of the parent antibiotic did not lead to complete mineralization. A part of the organic carbon remained in the solution, most likely in the form of transformation products or smaller organic molecules.

### 7.2 Carbamazepine (CBZ)

CBZ is an extensively studied pharmaceutical which is commonly present in water bodies and is comparatively difficult to remove due to its stable molecular structure. CBZ is degraded using g-C_3_N_4_-based photocatalytic system through various photodegradation pathways. Çako *et al.* studied CBZ degradation using a ZnFe_2_O_4_/(P,S)-g-C_3_N_4_/2-D TiO_2_ photocatalyst and identified six transformation products by LC-MS. CBZ degradation mainly involved electron-transfer oxidation, hydroxylation, deamidation, and ring transformation.^416^ The degradation resulted in formation of 10,11-epoxy-CBZ and 10-hydroxy-CBZ as intermediates, which were further oxidized into smaller products, including acridine and acridone. O_2_˙^−^ and ˙OH were mainly involved in photocatalysis, while SO_4_˙^−^ also contributed when persulfate was added. Moreover, >99.5% CBZ degradation was achieved within 15 min, whereas TOC removal reached only 74% after 120 min. This difference demonstrates that rapid disappearance of the parent CBZ molecule did not correspond to equally rapid mineralization.

### 7.3 Bisphenol A (BPA)

As previously mentioned, BPA is a commonly occurring endocrine disruptor, which can form several intermediates during photocatalytic degradation. In a representative study, g-C_3_N_4_/Bi_4_O_5_Br_2_ photocatalyst achieved 98% BPA degradation within 80 min, and the TOC removal reached 78.8%, showing that BPA was strongly degraded but not completely mineralized.^417^ The degradation pathway involved hydroxylation, bond cleavage, ring opening, and formation of smaller intermediates before further oxidation, however no toxicity assessment was reported in this study. In another study, Geleta *et al.* studied BPA degradation using CaCN_2_-template-modified g-C_3_N_4_ (MCN-0.25) under visible light.^418^ LC-MS analysis was used for identification of eight transformation products and suggested two main degradation pathways. The first pathway showed that the BPA was hydroxylated to form monohydroxylated BPA (*m*/*z* 243.10), which was then converted into benzoic acid (*m*/*z* 121.03) and smaller organic acids. Whereas, in the second pathway, ROS attacked the C–C bond connecting the aromatic rings of BPA, causing bond cleavage and the formation of smaller intermediates. EPR studies showed that O_2_˙^−^ was the dominant reactive species, while ˙OH and h^+^ also contributed to BPA degradation. ECOSAR analysis showed that most BPA transformation products were predicted to have lower acute and chronic toxicity than BPA. However, P4 showed higher predicted toxicity toward fish and *Daphnia*. These results shows that BPA degradation can sometimes form products with higher toxicity.

Complete mineralization is still difficult in g-C_3_N_4_ photocatalysis. Some toxic intermediates may remain even after the complete removal of pollutant. Therefore, TOC and toxicity should be checked along with degradation efficiency to confirm the safety of treated water. Moreover, longer irradiation time, better ROS production, and heterojunction engineering have been widely explored to improve mineralization.^419–421^ For example, Bi *et al.* demonstrated the successful synthesis of 2-D/2-D g-C_3_N_5_/g-C_3_N_4_ heterojunction (CNH) for the degradation of TC in wastewater and reported the improved TOC mineralization rate for CNH, as compared to pure g-C_3_N_5_ and g-C_3_N_4_.^421^ The author correlated the higher mineralization rate for S-scheme CNH photocatalyst with improved charge separation and ROS generation, which facilitated the oxidation of TC intermediates into smaller molecules.

## 8 Challenges and limitations

### 8.1 Charge recombination and limited charge utilization

g-C_3_N_4_ has emerged as a promising semiconductor photocatalyst for environmental remediation due to its green and metal-free nature. However, it still faces some limitations. Rapid charge recombination and low quantum yield remain the bottleneck for the use of g-C_3_N_4_ in practical applications.^424,425^ After light absorption, e^−^ are excited to the CB, leaving h^+^ in the VB. These charge carriers need to reach the catalyst surface to take part in photocatalytic reactions. However, many electrons and holes recombine or become trapped before reaching the surface. As a result, fewer charge carriers are available for ROS generation and pollutant degradation. Sabir *et al.* also provided numerical evidence for charge recombination in g-C_3_N_4_.^225^ TRPL results showed that the radiative recombination contribution was 54.4% for pristine g-C_3_N_4_ and decreased to 49.9% after forming the Cu-MOF/g-C_3_N_4_ heterojunction. Fs-TAS analysis also showed a 26.6 ps electron–hole recombination process related to trap states in pristine g-C_3_N_4_. These results show that charge recombination and trapping can reduce the number of charge carriers available for photocatalytic reactions. In a recent study, pristine g-C_3_N_4_ showed an apparent quantum yield of only 0.153%.^426^ The low quantum yield was mainly related to charge recombination, which reduces the number of photogenerated charge carriers available for surface reactions.

### 8.2 Activity–stability trade-off in modified g-C_3_N_4_

Although, many strategies, such as doping, co-doping, heterojunction formation, and defect engineering, have been developed to suppress charge recombination, however these are not without limitations. Excessive doping may result in formation of recombination centers, distort the crystal lattice, or reduce photocatalytic stability.^427,428^ For example, Phoon *et al.* reported that the GCN-F0.75 photocatalyst exhibited higher charge-transfer resistance than GCN-F0.50, which was attributed to the formation of recombination centers caused by excessive F doping.^249^ Likewise, He *et al.* reported that the excessive C-doping and defect introduction could disturb the graphitic framework of g-C_3_N_4_, leading to structural disorder and a decline in photocatalytic activity.^429^ Similarly, heterojunction formation requires complex synthesis route, and may suffer from poor interfacial contact.^299,302^ In a representative study, Qin *et al.* reported that low-mass interfacial contacts and poor charge-transfer efficiency severely limit the performance of semiconductor heterojunctions.^430^

### 8.3 Incomplete mineralization and toxicity of transformation products

As previously discussed in Section 7, most of the studies are focused on pollutant removal rather than complete mineralization and toxicity assessment of transformation products.^431–433^ The products formed during degradation are not always safer than the original pollutant. Some may remain toxic or have a greater tendency to accumulate in environment.^431,434^ For example, Wang *et al.* reported higher bioaccumulation factors for most TC intermediates compared with TC itself.^435^ A similar concern was reported for CBZ. Its predicted ChV for fish was 0.74 mg L^−1^, but the value dropped to 0.04 mg L^−1^ for TP 253B, showing higher predicted chronic toxicity.^436^ Another product, TP 222 had a high log *K*_ow_ of 4.63, which points to a possible bioaccumulation risk. Therefore, it is necessary to evaluate the toxicity of intermediates, as well as degradation products to ensure that the treatment does not produce more harmful compounds.

### 8.4 Real wastewater and continuous-flow operation

In most of the studies, ultrapure deionized water is used for photocatalytic degradation experiments containing a single pollutant. However real wastewater contains complex matrices of inorganic ions, natural organic matter, and several contaminants which can significantly reduce photocatalytic efficiency.^437–440^ For example, Cristaudo *et al.* found the lowest CBZ degradation rate in brackish water containing 1704.49 mg L^−1^ Cl^−^, 241.53 mg L^−1^ SO_4_^2−^, and 10.58 mg L^−1^ organic carbon due to matrix effects such as radical scavenging and light attenuation.^436^ These results indicate that degradation efficiency obtained in ultrapure water, may not be attained in real water.

Another challenge is that most laboratory studies are performed in batch reactors. Batch systems allow enough time for the pollutant to react with the photocatalyst but catalyst recovery and scale-up can be difficult.^441^ On the other hand, in continuous-flow systems, the contact time depends on the flow rate and reactor volume. A higher flow rate can shorten this time and may reduce pollutant removal.^442^ For example, Jiménez-López *et al.* studied immobilized g-C_3_N_4_ in a fixed-bed continuous-flow reactor for SMX removal and found that 100% SMX was removed within 90 min, however the same material under continuous operation conditions achieved approximately 80% SMX removal and that too at steady rate.^443^

### 8.5 Lack of standardized experimental protocols

Experimental conditions such as catalyst loading, CEC concentration, light source, light intensity, and pH vary widely across studies on g-C_3_N_4_ photocatalysis.^100,444^ These experimental conditions have a profound impact on the observed degradation efficiency of a photocatalytic system. For example, Ebenezer and Gandhimathi found that TC degradation by g-C_3_N_4_ decreased from 79.12% to 30.5% when its concentration increased from 5 to 40 mg L^−1^.^445^ Solution pH can also affect the reported activity. For example, TC degradation by pristine g-C_3_N_4_ increased from 83.5% at pH 4 to 97.8% at pH 10.^446^ Likewise, catalyst dose is another important factor that influence the degradation efficiency. A 2025 systematic review examined 60 g-C_3_N_4_-based studies on antibiotic degradation. Catalyst doses across the studies ranged from 0.02 to 4 g L^−1^. Importantly, the review found examples where increasing catalyst dose initially increased antibiotic removal, but further increases caused the efficiency to fall.^444^ Due to these variations, the fair comparison between different studies become very difficult.^440,447^ Therefore, the experimental conditions used in each study should be reported clearly.

### 8.6 Catalyst deactivation and long-term stability

Catalyst deactivation and long-term stability are important concerns for the practical application of g-C_3_N_4_-based photocatalysts. Pollutants and their degradation products can remain on the catalyst surface during treatment.^448,449^ This can block active sites and reduce the available surface for photocatalytic reactions. Long-term use may also cause changes in the catalyst itself, such as dopant leaching, changes in surface composition, or structural deterioration.

Cristaudo *et al.* recently studied the reuse of P/In-modified g-C_3_N_4_ for three photocatalytic cycles.^436^ The photocatalytic system retained its efficiency for CBZ degradation, however H_2_O_2_ production decreased by about 15% in the third cycle. The ICP-OES analysis revealed the release of In and P during reuse. The total amount released after three cycles was about 0.6% of the loaded In and 3.5% of the loaded P. The surface In content measured by XPS showed decreased from 1.4 at% to 0.9 at%.

Surface fouling can also limit the long-term performance of g-C_3_N_4_-based systems. Qi *et al.* operated a g-C_3_N_4_-modified membrane bioreactor for 60 days and found a fouling cake layer of 4.97 µm when the membrane was used in the dark.^449^ However, the cake-layer thickness decreased to 2.46 µm under visible light irradiation, which is corelated with photocatalytic degradation of organic foulants and lower accumulation of extracellular polymeric substances.

## 9 Future perspectives and research directions

The following subsections discuss emerging trends and future research needs for g-C_3_N_4_-based photocatalysts.

### 9.1 AI-assisted design of g-C_3_N_4_ photocatalysts

Machine learning (ML) is now being explored for the design and optimization of g-C_3_N_4_ photocatalysts. It can help in finding relationships between synthesis conditions, material properties and photocatalytic performance. Different models have been used for this purpose, including random forest, gradient boosting and neural networks.^450,451^

Yan *et al.* used ML for the screening of g-C_3_N_4_-based heterojunctions.^452^ Six different models were tested. CatBoost performed best, with an *R*^2^ of 0.9707 and RMSE of 0.0471. Band gap, formation enthalpy and molecular weight of the second semiconductor were among the important factors identified by the model. Based on the screening results, PbWO_4_ g-C_3_N_4_ was selected for further study. The predicted TC-HCl degradation was 90.2%. The catalyst was then prepared in the laboratory and tested. It showed 99.8% degradation of 10 mg L^−1^ TC after 180 min under a 350 W xenon lamp. Kumari *et al.* applied RF, decision tree, SVM and neural-network models to a g-C_3_N_4_/CdS/MoS_2_ system.^453^ The *R*^2^ values obtained with RF and NN were 0.7014 and 0.6864. These two models predicted 83.5% and 83.7% MB degradation, respectively. The experimental value was 86% after 180 min in sunlight. Thus, the predicted values were close to the experimental result.

One problem with ML in photocatalysis is the available data. Experimental conditions differ greatly between published studies. Catalyst dose, pollutant concentration, pH and light conditions are not the same, while photon flux is often not given. This makes it difficult to build large and consistent datasets. More experimental data are therefore needed. The data should also be reported under clearly defined conditions. Most importantly, ML predictions should be tested experimentally before a new catalyst is considered successful. Future studies should focus on improving the available datasets and experimentally testing the materials suggested by ML models. Some recent studies using ML for the prediction and optimization of g-C_3_N_4_-based photocatalysts are summarized in Table 8.

**Table 8:** Recent examples of machine-learning-assisted prediction and optimization of g-C_3_N_4_-based photocatalysts

Photocatalytic system	Target pollutant	ML approach	Model performance	ML prediction/selection	Experimental result	Ref.
PbWO_4_/g-C_3_N_4_	TC	XGB, CatBoost, RF, DT, SVM, GBDT	CatBoost: *R*^2^ = 0.9707, RMSE: = 0.0471	PbWO_4_/g-C_3_N_4_ selected, 90.2% predicted degradation	99.8% TC, 10 mg L^−1^, 180 min	452
g-C_3_N_4_/BiVO_4_	Norfloxacin	ML integrated with experimental optimization	*R* ^2^ = 0.8558, RMSE = 0.0510	Optimum hydrothermal condition identified	94.41%, 10 mg L^−1^, 120 min; *k* = 0.02618 min^−1^	454
Self-template g-C_3_N_4_	RhB	MLP-ANN, RF	ANN: *R*^2^ = 0.9774, RF: *R*^2^ = 0.563	Self-template route selected	∼100%; *k* = 0.0613 min^−1^	455
g-C_3_N_4_/CdS/MoS_2_	MB	RF, DT, SVM, NN	RF: *R*^2^ = 0.7014, NN: *R*^2^ = 0.6864	83.5–83.7%	86%, 180 min sunlight	453

### 9.2 Single-atom catalysts

The single-atom catalysts (SACs), such as Cu, Pt, Pd, Mo, and Ag, have emerged as the most significant conceptual advances in modern heterogeneous catalysis.^456–460^ Their key advantages include nearly 100% atomic utilization, maximized efficiency of expensive metals, enhanced reaction selectivity, and unique electronic properties that drive higher intrinsic catalytic activity.^461,462^ The integration of SACs with g-C_3_N_4_ produces hybrid materials that display improved photocatalytic activity beyond those of individual component.^463^ For example, Peng *et al.* prepared a MXene/Cu-single-atom-g-C_3_N_4_ heterojunction for visible-light-assisted PMS activation.^464^ The system removed 88.76% CIP within 150 min and achieved more than 70% TOC removal with a rate constant of 0.68 min^−1^, which was 4.8 times higher than Cu doped g-C_3_N_4_/PMS/Vis. The catalyst also maintained more than 80% CIP removal after seven cycles. Furthermore, Cu was present at only 0.09 wt%, showing that a very small amount of atomically dispersed metal can strongly improve the activity. Wu *et al.* anchored Ru single atoms on flake g-C_3_N_4_ through Ru–N_4_ coordination.^465^ With only 0.34% Ru, the catalyst achieved nearly 100% TC removal within 5 min, with a rate constant of 0.236 min^−1^. The catalyst also maintained about 99% of its activity after four cycles. Therefore, future work should focus on the rational design and exploration of next generation SAC-g-C_3_N_4_ photocatalysts for selective and efficient removal of CECs.

### 9.3 Advanced mechanistic studies

Advanced *in situ* and *operando* characterization techniques, such as *operando* SEM, TEM, Raman, XPS, EPR, are needed for investigation of charge-transfer pathways and photocatalytic mechanisms in real-time. Combining real-time observations with theoretical calculations will provide deeper insight into interfacial charge transfer process, ROS generation, and structure–activity relationships, during photodegradation process. Moreover, the comprehensive understanding of catalyst evolution during photodegradation reaction will help in establishing reliable design principles with improves stability and efficiency for next generation photocatalytic systems.

### 9.4 Reactor design and catalyst recovery

Most work on g-C_3_N_4_ is still done in small batch reactors. The catalyst is usually added directly into the polluted water as a powder. This allows good contact with the pollutant, but creates another problem. The catalyst has to be collected again after treatment. Filtration and centrifugation can be used for this purpose, but these extra steps become difficult when a large amount of water is treated. Using too much catalyst is also not helpful because it can block or scatter the incoming light.^466^ Thus, future reactor designs should also consider how easily the catalyst can be collected and used again.

One way to deal with this problem is to attach g-C_3_N_4_ to a solid support. Jiménez-López *et al.* tested this idea by fixing g-C_3_N_4_ on alumina supports and using them for SMX degradation in a fixed-bed reactor.^443^ Around 80–90% SMX removal was maintained during continuous operation. This result is encouraging for the use of g-C_3_N_4_ in flow reactors. Still, fixing the catalyst on a support has some drawbacks. Part of its surface may become less available to the pollutant, and mass transfer can also become slower.

More work is now needed on reactor systems that are simple to operate and allow repeated use of the catalyst. Immobilized g-C_3_N_4_ should remain firmly attached to the support while still receiving enough light and contact with the polluted water. Future studies should also test these reactors with real wastewater for longer periods. Flow rate, residence time, light penetration, catalyst recovery, and coating stability will all be important when moving from laboratory experiments toward larger-scale water treatment.

### 9.5 Energy demand, solar utilization, and cost

The energy used during treatment will be an important issue when g-C_3_N_4_ photocatalysis is moved to a larger scale. Most laboratory studies use xenon, UV, or LED lamps. These lamps are useful for controlled experiments, but they also consume electricity. Future studies should therefore report the energy used along with pollutant removal. This will help in deciding whether a highly active catalyst is also practical for water treatment.

Sunlight could reduce the electricity needed for irradiation. This is one of the main advantages expected from visible-light-active g-C_3_N_4_. However, solar treatment has its own problems. Sunlight changes during the day and is also affected by weather and season. Therefore, future solar reactors need to make better use of the available light and should be tested for longer periods under outdoor conditions. Recent reviews on g-C_3_N_4_ also identify improved solar-light utilization and practical scale-up as areas that still need further work.^467,468^

Energy use should also be reported in a form that allows comparison with other treatment methods. Electrical energy per order (*E*_EO_) is useful for this purpose. It represents the electrical energy required to reduce a pollutant concentration by 90% in one cubic meter of water. One g-C_3_N_4_ study gives a clear example of why the light source matters. For a g-C_3_N_4_/PMS system, *E*_EO_ was 24.51 kWh m^−3^ per order using a 400 nm LED, whereas it reached 2702 kWh m^−3^ per order with a xenon lamp.^469^ Although this was a PMS-assisted system, the large difference shows that the choice of lamp can strongly change the energy demand.

Cost should not be judged from catalyst price alone. Electricity, catalyst preparation and recovery, chemicals, and waste handling may all contribute to the final treatment cost. The cost is also different for each AOP. UV/H_2_O_2_ needs electricity for the UV lamp as well as a regular supply of H_2_O_2_. Ozone has to be produced during the treatment, which also uses electricity. In the Fenton process, Fe^2+^ and H_2_O_2_ are consumed and iron sludge is left after treatment. These costs cannot be ignored when g-C_3_N_4_ is compared with other treatment methods. Future studies should look beyond pollutant removal alone. The amount of energy and chemicals used, treatment time, and how long the catalyst can be reused are also important.

### 9.6 Scalable and sustainable production

Large-scale preparation is another area that needs more work. The usual thermal preparation of g-C_3_N_4_ is fairly simple. Melamine, urea, and other nitrogen-rich precursors can be directly heated to obtain the material.^25,470^ This simplicity is useful for scale-up. The situation is different when several modification steps are added. Acid treatment, exfoliation, templating, hydrothermal treatment, washing, and drying can make the synthesis longer. They also increase the use of water, solvents, and energy.^25,471^

The problem may become greater for doped g-C_3_N_4_, heterojunctions, and single-atom catalysts. Most of these materials are prepared and tested in small laboratory batches. Making a larger amount is not simply a matter of using more precursor. Heating and mixing may not remain uniform throughout a large batch. As a result, the material obtained at larger scale may not have exactly the same properties as the small batch. Furthermore, scale-up is not discussed in enough detail in many g-C_3_N_4_ studies. Most synthesis work is carried out with only a small amount of material. It would be useful to report how much final product is obtained from the starting precursor. The amount of water and solvent used during preparation also matters, particularly when several washing steps are involved. Heating and drying add further energy use. These details become important when the same method is tried with a larger batch. Work beyond the usual laboratory scale is therefore needed. A larger batch should not only give more material, it should also keep the properties that made the small batch effective. This is where simpler preparation methods may have an advantage.

## 10 Conclusions

g-C_3_N_4_ has emerged as one of the most promising metal-free photocatalysts for the removal of CECs due to its visible-light responsiveness, chemical stability, low cost, and tunable electronic structure. This review highlights recent advances in the fundamental understanding, synthesis, characterization, and modification of g-C_3_N_4_-based photocatalysts for environmental remediation. Strategies including doping, co-doping, heterojunction construction, carbon-based hybridization, and morphology and porosity engineering have significantly improved light harvesting, charge separation, and photocatalytic efficiency toward a wide range of pharmaceuticals, endocrine-disrupting compounds, and personal care products. Despite these achievements, challenges associated with charge recombination, catalyst stability, transformation-product toxicity, and performance under realistic wastewater conditions continue to hinder practical implementation. Future progress will rely on the development of rationally designed photocatalysts, deeper mechanistic insights obtained through advanced characterization techniques, and scalable synthesis approaches validated in real-world treatment systems. Addressing these challenges will be essential for utilization of g-C_3_N_4_-based photocatalytic technologies from laboratory studies to practical environmental applications.

## Author contributions

MG (particularly) wrote whole the manuscript, MSU revised the introduction and fundamental sections, MARA conceptualized & supervised, made all figures wrote – original draft, revised & edited.

## Conflicts of interest

There are no conflicts to declare.

## Data Availability

No primary research data, software, or code were generated or analysed as part of this review. All information discussed in this article is derived from previously published studies, which have been appropriately cited in the reference list.
